# Evaluation of Superconducting Magnet Shield Configurations for Long Duration Manned Space Missions

**DOI:** 10.3389/fonc.2016.00097

**Published:** 2016-06-08

**Authors:** Filippo Ambroglini, Roberto Battiston, William J. Burger

**Affiliations:** ^1^University of Perugia and INFN-Perugia, Perugia, Italy; ^2^University of Trento and TIFPA, Trento, Italy; ^3^FBK and TIFPA, Trento, Italy

**Keywords:** long duration manned space missions, active magnetic shielding, radiation protection, Monte Carlo simulation

## Abstract

A manned mission to Mars would present an important long-term health risk to the crew members due to the prolonged exposure to the ionizing radiation of galactic cosmic-rays. The radiation levels would largely exceed those encountered in the Apollo missions. An increase in the passive shielding provided by the spacecraft implies a significant increase of the mass. The advent of superconducting magnets in the early 1960s was considered an attractive alternative. The technology allows to generate magnetic fields capable to deflect the cosmic-rays in a manner analogous to the reduction of the particle fluxes in the upper atmosphere due to the Earth’s dipole magnetic field. A series of the three studies have been conducted over the last 5 years, funded successively by European Space Agency (ESA), the NASA Innovative Advanced Concepts (NIAC) program, and the Union European’s Seventh Framework Programme (FP7). The shielding configurations studied are based on high-temperature superconductors, which eliminate the need to operate with liquid helium. The mass estimates of the coils and supporting structure of the engineering designs are based on the current and expected near-future performance of the superconducting materials. In each case, the shield performance, in terms of dose reduction, is provided by a 3-dimensional Monte Carlo simulation, which treats in detail the electromagnetic and hadronic interactions of the galactic-cosmic rays, and the secondary particles they produce in the materials of the shield and spacecraft. A summary of the results of the studies, representing one of the most detailed and comprehensive efforts made in the field, is presented.

## 1 Introduction

**Figure 1:**
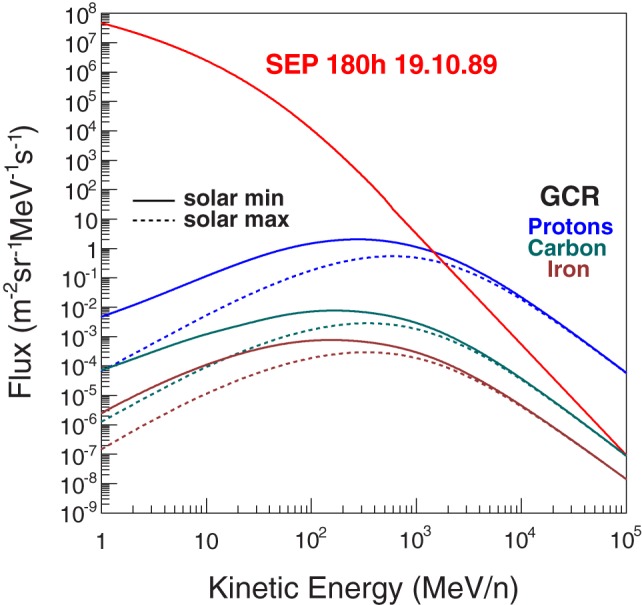
**The proton flux of a 10-day SEP event in October 1989, and the GCR proton, carbon, and iron nuclei fluxes for solar minimum and maximum periods (1)**.

The exposure to the ionizing radiation of galactic cosmic-rays (GCR) and solar energetic particles (SEP) is an important concern for the health of the crew for long duration interplanetary missions. Figure 1 shows the proton flux of a 10-day SEP event and the GCR fluxes for protons, carbon, and iron nuclei. The SEP events are characterized by the emission of high fluxes of lower energy particles, which may last on the order of hours or days. The GCR fluxes are modulated by the solar cycle characterized by alternating periods of maximum and minimum activity. Periods of maximum solar activity result in the decrease of the low energy GCR flux due to their interaction with the higher particle flux emitted by the Sun.

The radiation risk arises from the damage caused by the energy lost by the charged particles in human tissue. The mean energy loss rate in a material due to ionization is given by the Bethe-Block equation,

(1) $$\frac{\mathrm{dE}}{\mathrm{dx}}=4\pi N_{A}r_{e}^{2}m_{e}c^{2}\frac{z^{2}}{\beta^{2}}\frac{Z}{A}\left[\frac{1}{2}\ln\frac{2m_{e}c^{2}\beta^{2}\gamma^{2}T_{m}}{I^{2}}-\beta^{2}-\frac{\delta }{2}\right]$$

where *z* and β are the particle’s charge and velocity expressed in terms of the speed of light *c*. *Z* and *A* are the atomic number and mass of the material. The other terms in the equation are Avogadro’s number *N_A_*, the classical radius *r_e_* and mass *m_e_* of the election, the relativistic term $\gamma =\sqrt{1-\beta^{2}}$, the mean excitation energy *I*, the maximum kinetic energy energy lost in a collision with a free electron *T_m_*, and a density correction term δ.

The ionization losses depend on the characteristics of the material traversed and the properties of the ionizing radiation. The materials may be classified as sensitive, for example, electronics and human tissue, and inert, i.e., insensitive to the ionizing radiation. Passive shielding refers to the slow down and absorption of the charged particles in inert materials. Particles with high ionization rates, i.e., with high charge *z* and low velocity *β*, are responsible for high doses in sensitive materials, whereas they are shielded efficiently by a passive absorber.

The ionization of the lower energy SEP protons represents a short-term risk due to the very high flux of the low velocity protons. The GCR fluxes represent a longer term risk exposing the crew members to lower life expectancy due to radiation induced cancers.

In addition to the ionization losses, the GCR protons and nuclei are subject to inelastic nuclear interactions in the material traversed, which produce lower energy secondary charged particles and neutrons. The ionization loss in the inert materials of the spacecraft provides passive shielding if sufficiently thick to contain the primary particles and their secondaries. In the case of SEP events, the required thickness of the shielding material would limit the protection to a small volume of the spacecraft, a shelter that would be occupied during the duration of the event.

The appearance of superconducting magnets in the early 1960s presented an alternative, an active magnetic shield (2). A particle with charge *q* and vector velocity **v** is deflected in the plane perpendicular to the magnetic field by the Lorentz force,

(2) F=qv×B.

The particle moves in a circle in the deflection plane, with an angular deviation *θ* from the incident direction,

(3) $$\theta \approx \frac{\mathrm{BL}}{R}$$

where *B* is the magnetic flux density, *L* the length of the field region in the deflection plane, and *R* the magnetic rigidity, i.e., the particle’s momentum divided by its electric charge.

The high field flux densities of superconducting magnets may be used to create an active magnetic shield, where particle deflection in the magnetic field replaces the energy ionization loss in the passive shield material. The alternative is particularly attractive for GCR protons, since the dose due to secondary particle production remains significant for realistic passive shielding configurations. In principle, the presence of large field volumes, free of material, would significantly reduce the secondary production, with a corresponding reduction in the shield mass with respect to a passive shield of equivalent performance.

Several groups have presented active shield designs based on superconducting magnet configurations. The more detailed studies concern shield configurations composed of toroid magnets. The performances are quoted in terms of the GCR flux reduction (3, 4) or dose reduction (5). Unconfined field configurations have been proposed without detailed estimations of the shielding performance (6, 7). The proposed unconfined fields require important modifications of the spacecraft architecture.

## 2 Magnet Shield Configurations

Magnet shield configurations based on the high-temperature superconductors (HTSC) yttrium-barium-copper-oxide (YBCO) and magnesium diboride (MgB_2_) were evaluated in the ESA (8), NIAC (9), and the Space Radiation Superconducting Shield (SR2S)^1^ studies. The operating temperatures of the HTSC materials (~25 *K*) do not require the use of liquid helium, which represents a significant advantage in view of the technical difficulties encountered to guarantee the stability of the cryogenic system in space. A large volume magnetic shield operating with liquid helium was not considered technically feasible since it would require a significant extrapolation of current technologies.

The performance evaluations are based on the results obtained with 3-dimensional Monte Carlo simulations that propagate the charged particles in the magnet field, and generate interactions of the particles in the materials of the coils and support structures of the magnet shield. The material composition of the engineering shield designs, based on realistic extrapolations of existing technology, was used to describe the magnetic shields in the simulations. A brief description of the HTSC shields of the three studies follows.

### 2.1 ESA Study

YBCO superconductors are manufactured in the form of a 4-mm-wide tape with a thickness smaller than 0.2 mm. Multilayer cylindrical coils composed of YBCO tape may be used to form pure multipole fields by modulating the angle of the helical turns in successive tape layers (10). The magnesium diboride superconductors are produced in cable form; the individual MgB_2_ wire elements are brittle and require a rigid support.

**Figure 2:**
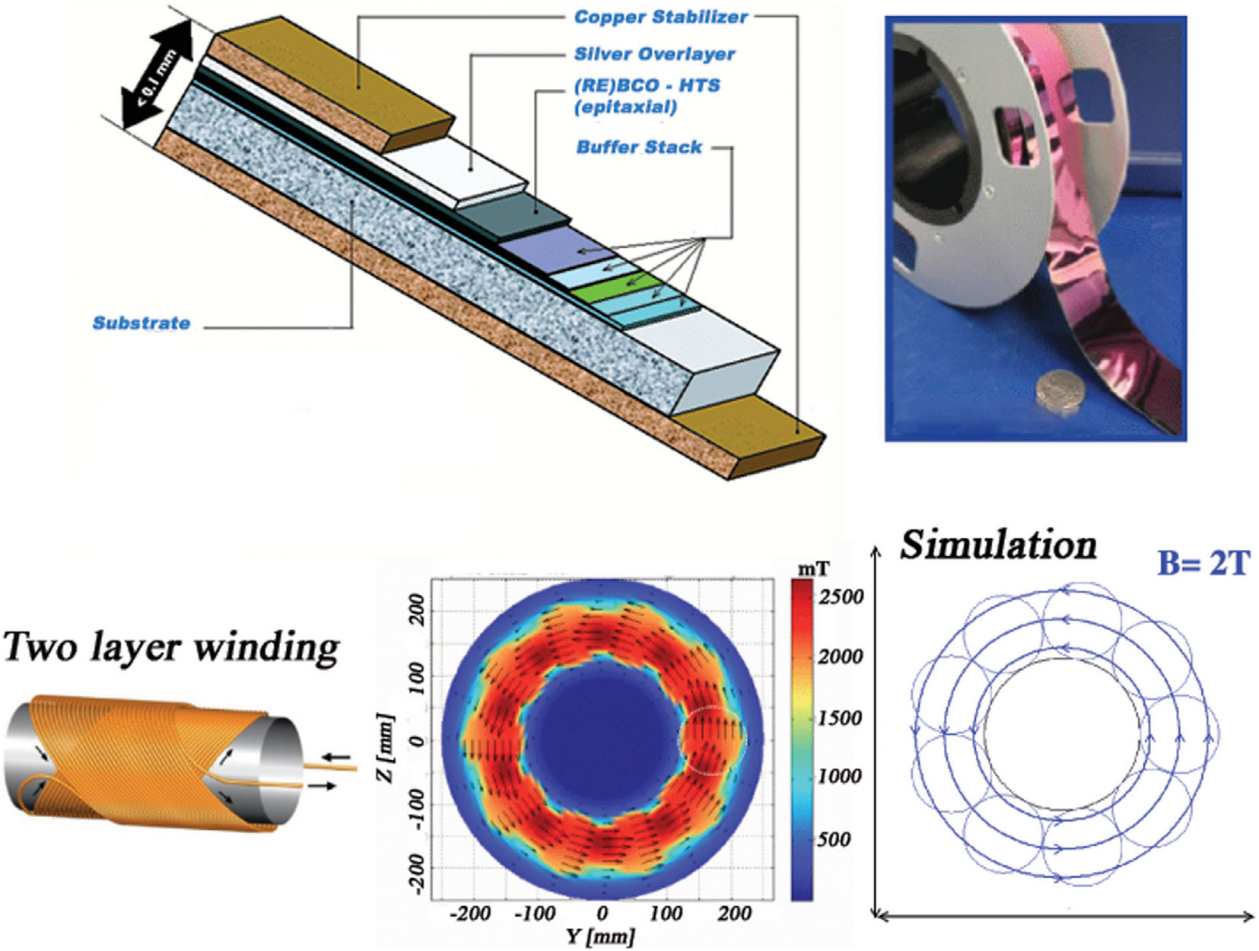
**The composition of the YBCO tape from SuperPower (top left) and a photograph of a 0.2-mm-thick tape produced by American Superconductor (top right)**. Two-layer winding used to produce the double-helix solenoid field (bottom left). The field map, from Advanced Magnet Lab (AML), of the toroidal field (8) around the habitat (bottom center) and the field used in the Monte Carlo simulation (bottom right).

Two toroidal field configurations were considered in the ESA study. Figure 2 illustrates the double-helix solenoid coil shield concept based on the YBCO superconductor. A dipole field is produced in each solenoid coil by reversing the current direction in the opposite-tilt-direction layers of the helical windings. The solenoid coils are oriented to produce a toroidal field around a 4-m-diameter cylindrical habitat in the simulation. The superposition of the fields of the 2-m-diameter coils results in a nearly homogeneous axial field with a BL ~4 Tm.

The second configuration of the ESA study was a racetrack coil toroid composed of MgB_2_ superconducting cable. The field integral of the 12 racetrack coil toroid is 4.9 Tm. The racetrack geometry is commonly used in high energy physics. The dimensions of the low-temperature superconducting racetrack toroid of the Atlas experiment at the Large Hadron Collider (LHC-CERN) are comparable with the dimensions required for a radiation shield in the space application.

### 2.2 NIAC Study

A modified version of the YBCO coil shield was developed in the NIAC study. The dipole field, obtained with the multiple layer winding, was abandoned in favor of a simpler, single orientation winding used to produce a solenoid field. The coil diameter was increased to 8 m and the field flux density reduced to 1 T, yielding a maximum field integral of 8 Tm at the center of the coil, and an average value of 6.3 Tm, taking into account the path length variation across the diameter of the cylindrical coil.

**Figure 3:**
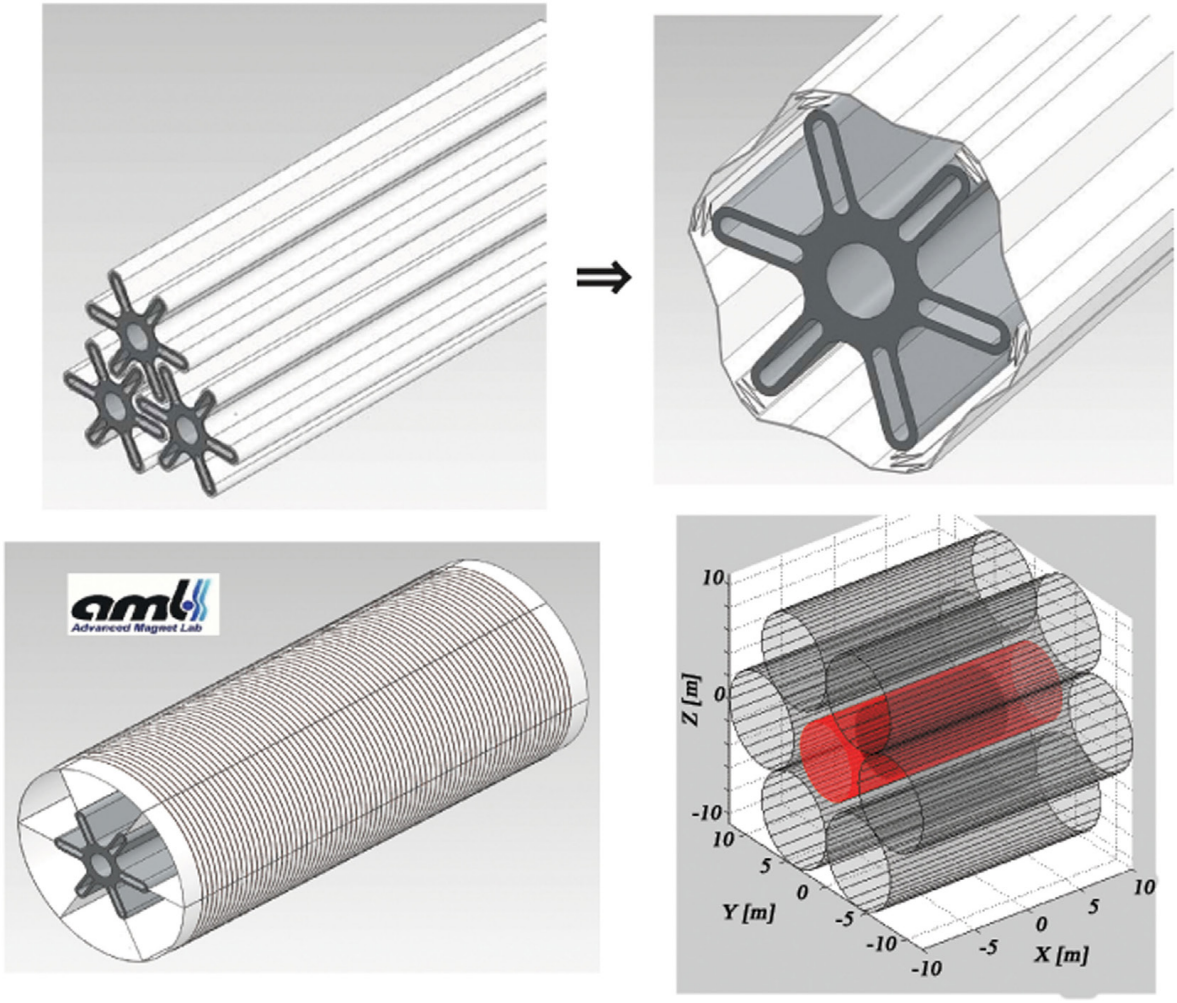
**The 6 + 1 extendable solenoid shield, developed by Advanced Magnet Lab (AML) in the NIAC study (9): coils packed for launch (top left), deployment in space (top right), the fully deployed 8-m-diameter solenoid coil (bottom left) and the final 6 + 1 configuration with the compensation solenoid (red) in the center (bottom right)**.

The reduction of the number of YBCO tape layers increases the flexibility of the coils, and allows a compact storage for launch. After deployment in space, the coils expand to their full diameter when the current is applied, due to the effect of the Lorentz force acting on the current flowing in the flexible coils. The fringe fields of the 20-m-long solenoids are compensated by a central solenoid coil concentric with the cylindrical habitat, which reduces the field inside the habitat to an acceptable level. The 6 + 1 extendable solenoid coil shield is illustrated in Figure 3.

### 2.3 SR2S Study

The SR2S consortium has chosen to pursue the racetrack coil toroid configuration with the magnesium diboride cable. A continuous-coil toroid, consisting of 120 racetrack coils, with a field integral of 8 Tm, protects a 4.5-m-diameter, 6-m-long cylindrical habitat based on the design of the ESA Columbus scientific module of the International Space Station.

**Figure 4:**
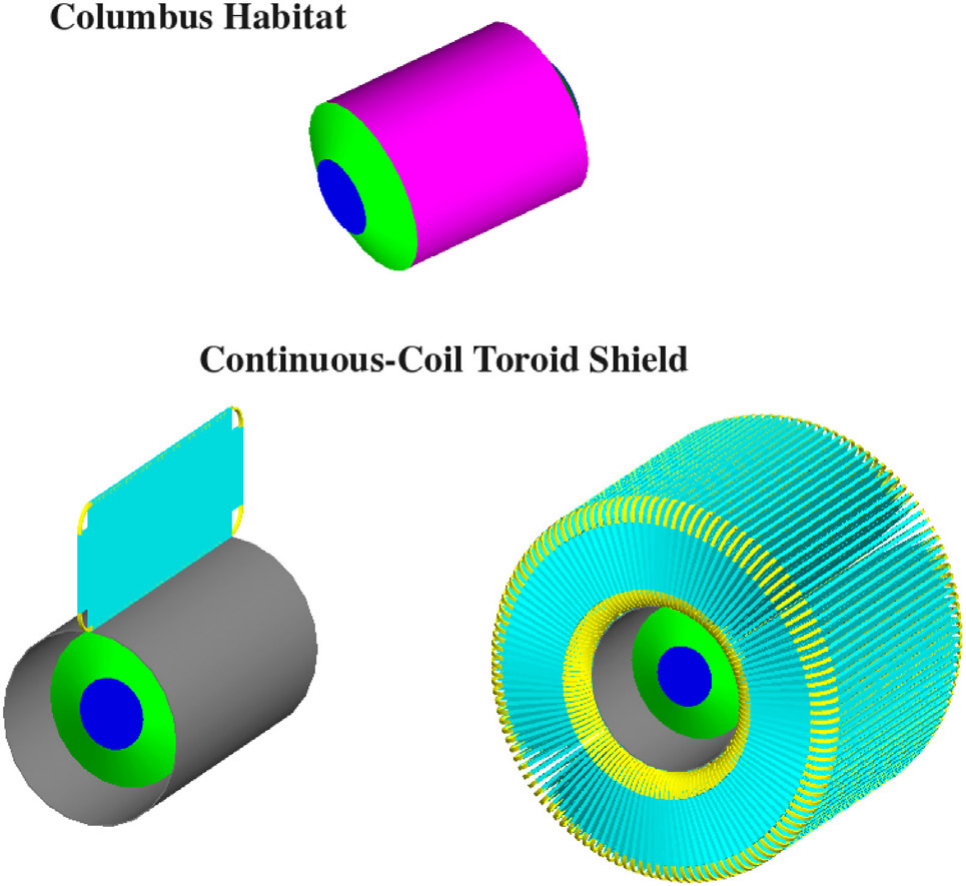
**The 4.5-m-diameter, 6 m long Columbus habitat (top), and the continuous-coil toroid shield of the S2RS study**. A single racetrack coil (lower left) and the 120 coil toroid (lower right) are shown with the Al-B4C support cylinder (gray) that surrounds the Columbus habitat (blue-green). Each coil (yellow) is supported by a 0.6-mm-thick KEVLAR sheath (cyan).

The habitat and the position of the coils around the habitat are shown in Figure 4. Each coil is surrounded by a 0.6-mm-thick KEVLAR support sheath. A 8.6-m-long support cylinder composed of a metal matrix composite material, aluminum-boron-carbide (Al-B4C), surrounds the habitat to support the Lorentz force acting on the coils in the direction of the habitat.

The multiplication of the number of coils in the continuous-coil design reduces the force acting on a single coil. The result is an overall reduction of the magnet shield mass and a greater mechanical tolerance for the toroid assembly.

## 3 Monte Carlo Simulations

A Geant3 (11) simulation was used for the ESA and NIAC studies. The FORTRAN code, which is a modified version the AMS Monte Carlo simulation program, was used for the study of Ref. (5). Geant3 performs particle propagation in magnetic fields and materials with a detailed treatment of electromagnetic interactions. Additional models have been implemented for hadron interactions of proton and He nuclei (Geant-FLUKA (12)). Nuclear cross sections (13) and fragmentation models (14) have been implemented in the AMS Geant3 simulation.

Geant3 is no longer supported by CERN since the early 2000s and has been progressively replaced in the scientific community by the C++ program Geant4 (15). A Geant4 version of the Geant3 simulation used for the radiation studies has been developed during the 2-year NIAC study. The same methods for the dose determination and sampling are implemented in the two simulations. The Geant4 simulation was used for the SR2S study.

### 3.1 Dose Determination

The ionization energy losses (Equation (1)) recorded during the track propagation *dE_i_* are converted to an dose equivalent ϵ*_i_* (Sv) by multiplying the absorbed dose $\frac{dE_{i}}{m}$ (Gy), where *m* is the mass of the volume considered, by the quality factor *Q*(*L*) defined by the unrestricted linear energy transfer in water *L* (keV/μm):

(4) $$\epsilon_{i}=Q\left(L\right)\cdot \frac{{\mathrm{dE}}_{i}}{m},$$

with

(5) $$L=\frac{{\mathrm{dE}}_{i}}{\mathrm{dx}}$$

and

$$Q\left(L\right)=\left\{\begin{array}{ccc} 1 & \text{for} & L\leq 10\\ 0.32\cdot L-3.2 & \text{for} & 10<L<100\\ 300/\sqrt{L} & \text{for} & L\geq 100 \end{array}\right\}$$

The total dose equivalent *d_z_*(*E_j_*) for an exposure time *t* due to GCR of charge *Z* and kinetic energy *E_j_* is the sum of the dose equivalents recorded for *N_j_* incident particles generated with the flux *f_z_*(*E_j_*) (cm^−2^sr^−1^s^−1^MeV^−1^), in the energy interval Δ*E_j_* (MeV), over the acceptance *A* (cm^2^sr):

(6) $$d_{z}\left(E_{j}\right)=\sum_{i}\epsilon_{i}\cdot \frac{A}{N_{j}}\cdot f_{z}\left(E_{j}\right)\cdot {\mathrm{\Delta E}}_{j}\cdot t.$$

The total GCR dose *D* is obtained by extending the generation over suitable ranges in charge and kinetic energy. The contribution from charge *Z* is

(7) $$d_{z}=\sum_{j}\;{\left[\sum_{i}\epsilon_{i}\right]}_{j}\cdot A\cdot \sum_{j}\;\frac{f_{z}(E_{j})\cdot {\mathrm{\Delta E}}_{j}}{N_{j}}\cdot t$$

and the total dose equivalent, including all charges up to Ni, $D=\sum_{z=1}^{z=28}\;d_{z}$.

Three terms contribute to the estimated dose level. The kinetic energy spectra *f_z_*(*E_j_*) that are taken from the CREME 2009 GCR model (1) with an energy range from 1 to 10^5^ MeV/n. A significant decrease of flux below 1 GeV/n is observed during the solar maximum (Figure 1). A solar cycle lasts 14 years divided roughly in equal length periods of solar maximum and minimum activity. The GCR spectra at solar minimum are used for the performance evaluation of the shield configurations. The second term *t* represents the mission length, or more precisely the duration of the exposure to the GCR flux in interplanetary space. Finally, the magnitude of the third term Σ*_i_*ϵ*_i_* depends on the effectiveness of the passive and active shielding elements.

### 3.2 Dose Sampling

**Figure 5:**
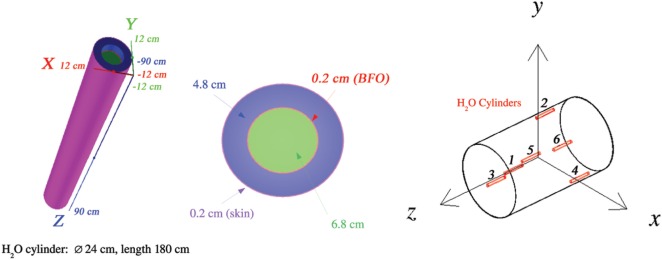
**At the left, the cylindrical water volume used to compute the dose of the skin and blood-forming organs (BFO)**. The body dose refers to the full volume of the 24-cm-diameter, 180-cm-long cylinder (81.4 kg). At the right, the positions of the six water cylinders in the cylindrical habitat.

The human body is represented in the simulations as a 24-cm-diameter, 180-cm-long water-filled cylinder. The cylinder is subdivided to define the regions used to compute the dose associated with the skin and blood-forming organs (BFO), respectively, the first 2 mm at the surface of the cylinder and a 2-mm-thick layer located a depth of 5 cm from the cylinder surface (Figure 5). The body dose refers to the ionization losses recorded in the full volume of the cylinder.

Figure 5 shows the positions of the six water cylinders used to record the dose levels in the cylindrical habitat. Three cylinders are present in each side ($\pm \hat{z}$) of the habitat. Cylinders 1 and 6 are aligned along the longitudinal axis; cylinders 2–5 are placed near the habitat cylindrical shell.

Secondary neutrons and gammas are generated and tracked. The non-ionizing, neutral particles do not contribute directly to the dose. The neutrons produce charged secondaries due to nuclear interactions in the water cylinder or surrounding materials, which may contribute to the dose recorded in the water cylinders, e.g., energetic protons from elastic scattering on hydrogen nuclei. Gammas contribute via charged secondaries produced in electromagnetic interactions.

## 4 ESA Study Results

**Figure 6:**
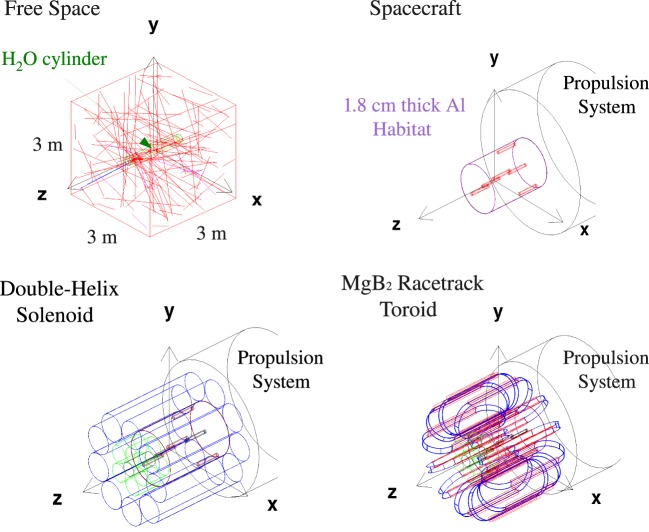
**The principal configurations evaluated in the ESA study (8): free space (upper left), spacecraft (upper right), the double-helix solenoid coil shield (lower left), and the racetrack coil toroid shield (lower right)**.

The principal configurations evaluated in the Geant3 simulation in the ESA study were free space, the spacecraft, and the two toroidal field magnetic shields (Figure 6). The free space results were compared to previously published results. The dose levels of the active magnetic shield configurations were compared to the free space, habitat, and spacecraft doses.

### 4.1 Free Space

The free space doses were evaluated at solar minimum and maximum. A single water-filled cylinder is placed at the center of a 3 m × 3 m × 3 m vacuum-filled cube. The incident GCR nuclei are generated uniformly on the surface of the cube.

The annual dose equivalents for protons, He nuclei, and *Z* > 2 nuclei are reported in Table 1. The free space results were obtained with a sample of 10 M protons and He nuclei, and 50 M, *Z* > 2 nuclei, i.e., with respectively, 0.2 M/m^2^ and 0.9 M/m^2^ incident particles on the generation cube. The particle densities are factors 2 and 15–25 higher than those used for the shielding configuration results reported for the two charge groups. The effect of the generation statistics on the quoted free space doses is negligible (~1%).

**Table 1:** **Annual free space dose equivalents (cSv/y)**.

	Solar minimum			Solar maximum		
Z	Skin	BFO	Body	Skin	BFO	Body
1	10.8	11.3	11.1	5.5	5.6	5.6
2	5.3	5.2	5.1	2.9	2.8	2.7
3–10	35.9	22.2	11.8	22.1	14.8	6.8
11–20	38.4	16.6	14.8	23.1	11.2	9.2
21–28	27.3	7.1	8.7	17.4	5.1	5.8
Total	117.7	62.4	51.5	71.0	39.5	30.1

The BFO annual dose equivalent of 62.4 cSV at solar minimum is compatible with values in the range between 58 and 70 cSv quoted in Ref. (16). The BFO annual dose at solar maximum, 35 cSv (16), is 10% lower than the 39.5 cSv reported in Table 1.

**Figure 7:**
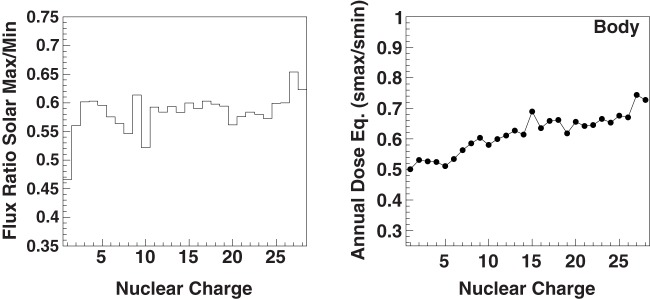
**The ratio of the energy-integrated cosmic-ray nuclei fluxes between solar maximum and minimum (1) (left) and the corresponding ratio of the free space body dose equivalent (right)**.

The ratio of the flux and dose reductions, between solar maximum and minimum, of the individual nuclei are shown in Figure 7. The local peak in the ratio at *Z* = 9 is explained by the absence of an anomalous GCR flux contribution for this element in the charge range 6 ≤ *Z* ≤ 10 (1). The free space body dose equivalent is reduced by ~40% at solar maximum.

### 4.2 HTSC Toroidal Field Configurations

Two toroidal field configurations based on the YBCO and MgB_2_ superconductors were studied. The axial field of the toroid shield configurations is adapted to the classical, cylindrical spacecraft geometry imposed by launch constraints. The confined field simplifies the design since a significant fringe field in the habitat would be unacceptable for the crew, and may affect vital operations of the spacecraft.

The YBCO double-helix solenoid and the MgB_2_ racetrack coil toroid shield configurations are shown in Figure 6. The spacecraft structures are composed of a 4-m diameter, 5.5-m-long cylindrical habitat surrounded by 1.8-cm-thick aluminum, and the propulsion system. The propulsion system is represented in the simulation by a solid 1.16-m diameter, 6-m-long aluminum cylinder. Air is present in the interior of the habitat.

The orientation of the axial toroidal field in the *xy* plane deflects charged particles with a momentum $+p_{z}\hat{z}$ in the direction of the habitat. The diameter of the aluminum cylinder of the propulsion system exceeds the radial extension of the field volume in order to eliminate GCR entering the field volume from the $-\hat{z}$ direction.

**Figure 8:**
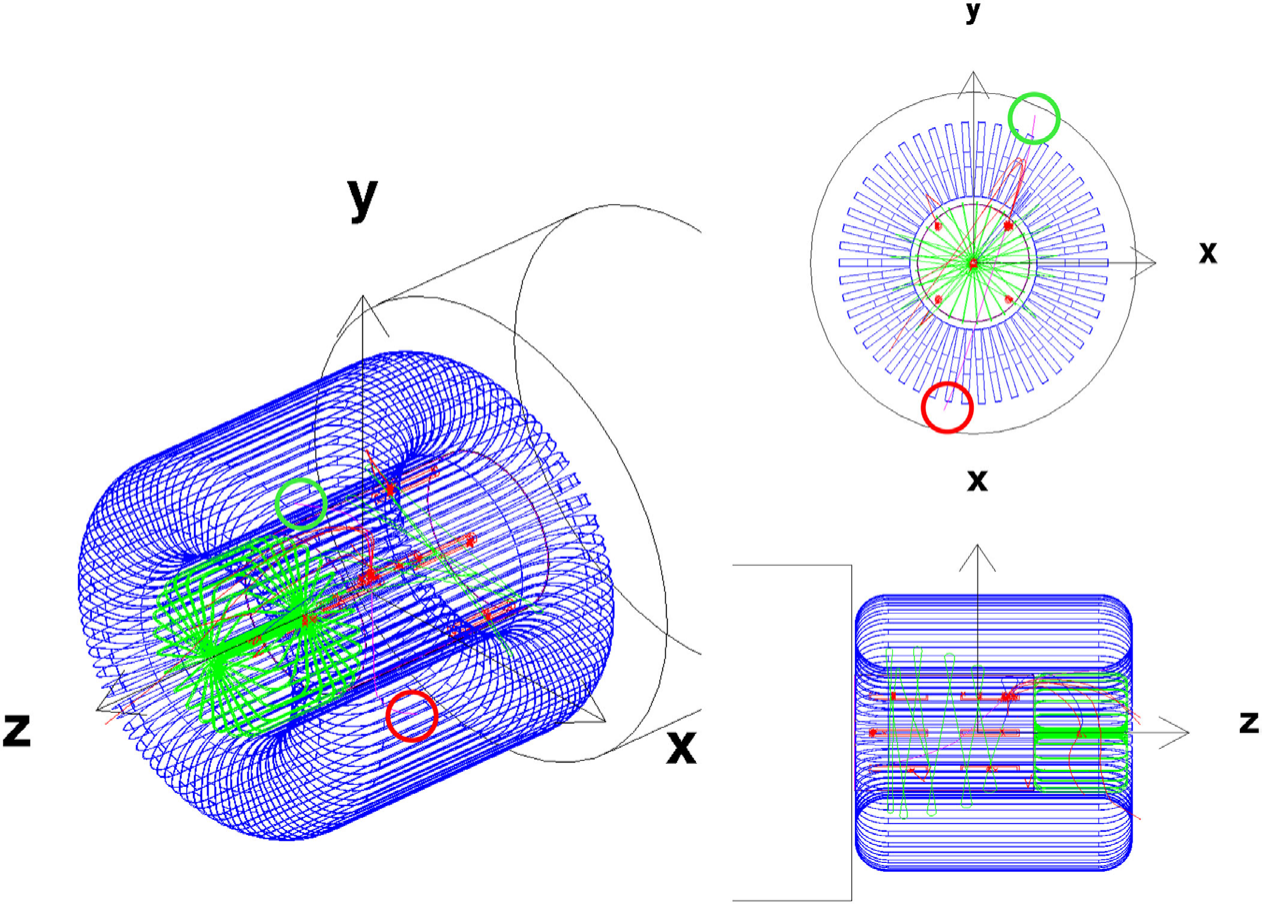
**A GCR carbon nucleus event with multiple ionization losses (red crosses) in the water cylinders**. The carbon nucleus (magenta) interacts in a water cylinder producing secondary pions (blue), which decay into muons (green), and neutrinos (not displayed). The incident (exit) direction of the C nucleus (magenta) is indicated by the red (green) circle.

The performance of the low-temperature superconducting (LTSC) toroid shield of Ref (5) was re-evaluated in the ESA study. The LTSC toroidal configuration is shown in Figure 8, with the particle tracks and recorded ionization losses in the water cylinder caused by an incident carbon nucleus.

The incident 2.85 GeV/n GCR carbon nucleus travels in the $\left(+\hat{x},+\hat{y},+\hat{z}\right)$ direction, with $\frac{p_{z}}{p}=0.83$, and is deflected toward the habitat where it interacts in a water cylinder creating secondary pions. One of muons from a pion decay is deflected across the habitat as it traverses the spacecraft. The event was selected among the millions generated by demanding that an ionization loss recorded in the water cylinders was produced by the recoil of an oxygen nucleus.

**Figure 9:**
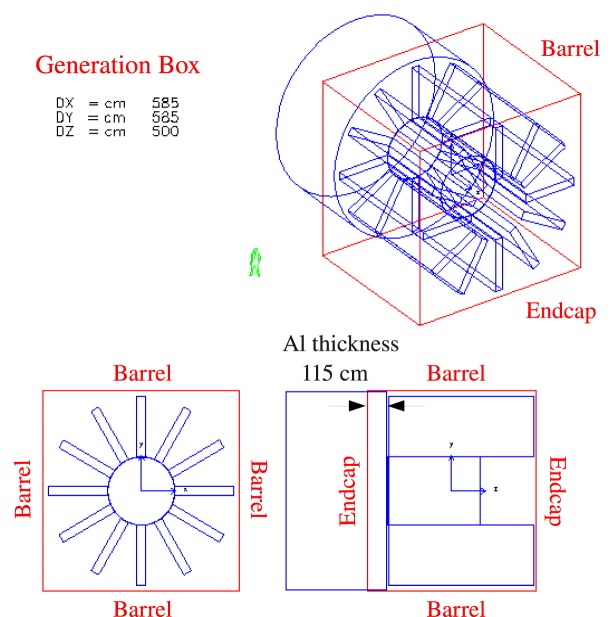
**The generation surface, a 11.7 m × 11.7 m × 10.0 m cube, used in the Geant3 simulation for the MgB_2_ racetrack coil toroid of the ESA study**. The effective thickness of the Al cylinder (propulsion system) is defined by the relative position of the spacecraft in the generation cube. The quoted performances for the three studies refer to dose levels produced by GCR generated on the four lateral sides of the cube (barrel region), which surround the active magnetic shields.

A second, smaller LTSC toroid (green) is present on the $+\hat{z}$ side of the spacecraft in Figure 8. A similar scheme for the double-helix solenoid coil toroid configuration was implemented in the simulation (Figure 6). However, no corresponding engineering study was made for the smaller toroid. Since the aim of the ESA study was to provide an assessment of the performance taking into account the contributions of the field and passive elements of realistic active magnetic shield designs, the performance evaluation was limited to the acceptance of the barrel region, defined by the lateral sides of the generation box, illustrated in Figure 9.

The double-helix solenoid coils are represented in the simulation by eight layers of 90 μm copper, which has a charge density, and radiation length very close to the average values of the materials of the YBCO superconducting tape. The coil support is represented by 2-mm-thick carbon cylinder.

The MgB_2_ superconducting coil is composed of copper, aluminum, Ti, and MgB_2_ layers with thicknesses of 0.30, 1.65, 1.50, and 1.55 cm, respectively. A 5-mm-thick aluminum frame support surrounds the coils.

The results of the performance evaluation are presented in Table 2. For each magnet shield configuration, the field integral BL, the total mass in the Monte Carlo, the total estimated mass of the corresponding engineering design, and the annual BFO dose equivalent for the barrel region acceptance are reported. The annual BFO dose equivalent limit for low Earth orbit (LEO) is 50 cSv (16).

**Table 2:** **A comparison of the ESA study active magnetic shields and habitat dose levels**.

Configuration	BL (Tm)	M_sim_ (t)	M_eng_ (t)	BFO (cSv/y)
LTSC toroid	19	12.5	–	19.2 ± 1.1
HTSC double-helix solenoid coil toroid	4	11.2	47.2	29.3 ± 2.3
HTSC racetrack coil toroid	4.9	28.0	46.4	26.7 ± 1.1
Habitat	–	4.54	–	35.6 ± 1.8

*The first mass quoted refers to the shield mass present in the Monte Carlo simulation. The second is the mass estimate from the engineering designs. The annual BFO dose equivalents refer to the GCR flux at solar minimum (1) and the barrel region acceptance (Figure 9). The quoted uncertainties represent the root-mean-square deviation of the average dose recorded in the six water cylinders (Figure 5)*.

The two HTSC configurations have a comparable performance providing a ~25% reduction of habitat dose level. The smaller dose of HTSC racetrack coil may be attributed to the higher *B* and the larger shield mass in the simulation, i.e., a larger contribution from passive shielding.

The dose estimates of the HTSC double-helix solenoid coil toroid where obtained with 37 M protons and He nuclei, and 7 M, *Z* > 2 nuclei. The corresponding numbers for the HTSC racetrack coil toroid are 22.5 M protons and He nuclei, and 4 M, *Z* > 2 nuclei. The quoted uncertainties in Table 2 represent the root-mean-square of the average dose recorded in the six cylinders, which reflects both the uniformity of the dose distribution in the habitat and statistical fluctuations.

The LTSC configuration BFO dose equivalent, obtained with a factor ~4.75 higher BL, is 40–50% lower than the values for the HTSC double-helix solenoid and racetrack coil shield configurations. The quoted shield mass of the LTSC configuration is the mass of the coils composed of aluminum.

The simulation and engineering masses are presented in Table 2 to indicate the level of accuracy of the HTSC shield descriptions in the simulation. The results presented in Table 2 are indicative. They represent the status attained at the end of the 1-year study. A definitive evaluation requires a complete description of the material of the active magnetic shield in order to take into account both passive and active shielding contributions to the dose reduction.

The results for the LTSC toroid over the full acceptance, 27.2 ± 1.5 cSv, including the shielding contributions of the second toroid and propulsion system, may be compared to the annual BFO dose equivalent of 18–33 cSv reported previously with the same simulation program (5). The range in the estimated dose reported in Ref. (5) reflects the estimated uncertainty in the GCR flux. The LTSC result of the ESA study was obtained with 37 M proton and He nuclei, and 15 M, *Z* > 2 nuclei.

### 4.3 Field and Coil Contributions

The explicit contribution of the coils to the dose reduction was made with a preliminary description of the double-helix solenoid coil, represented in the simulation by 1-cm-thick aluminum. The dose equivalents of the double-helix (DH) solenoid shield with BL = 2 Tm are compared in Table 3 to the corresponding spacecraft doses, and a second DH solenoid shield dose estimate made without the coil material in the simulation.

**Table 3:** **Effect of the magnet material on the annual dose equivalents (cSv/y) at solar minimum**.

	Spacecraft			DH with Al coils			DH without Al coils		
Z	Skin	BFO	Body	Skin	BFO	Body	Skin	BFO	Body
1	18.4	13.5	14.1	19.4	13.6	14.4	17.7	12.6	13.3
2	8.3	5.9	5.9	8.2	5.9	6.0	7.7	5.2	5.6
3–10	19.9	13.2	6.6	12.8	8.8	4.5	17.6	11.9	5.8
11–20	17.3	9.4	7.1	9.5	5.5	4.2	14.7	7.6	6.3
21–28	10.3	3.2	3.1	4.1	1.4	1.4	7.5	3.1	2.7
Total	74.2	45.2	36.8	54.0	35.2	30.5	65.2	40.4	33.7
Fraction of spacecraft dose				0.73	0.78	0.83	0.88	0.89	0.92

*The double-helix solenoid shielding configuration doses correspond to the full acceptance, including the endcap regions*.

The magnetic field and the material of the coils reduce the spacecraft BFO dose equivalent by 22%, with equal contributions to the reduction from the field and the passive shielding of the coils. The reduction of the spacecraft dose equivalents (~10%) due to the field alone is observed for all charge groups. The presence of the coils produces an additional reduction of the *Z* > 2 nuclei doses, and an increase of the proton and He nuclei doses due to the secondary particles produced in the coil material.

The results presented in Table 3 were obtained with 25 M (spacecraft), 73 M (with coils), and 41 M (without coils) protons and He nuclei. The corresponding numbers for *Z* > 2 nuclei are 3.7, 10.9, and 3.6 M, respectively. The uncertainties in the quoted total doses, based on the root-mean-square deviation of the average dose recorded in the six water cylinders, are 15% (spacecraft), 10% (with coils), and 15–20% (without coils).

## 5 NIAC Study Results

**Figure 10:**
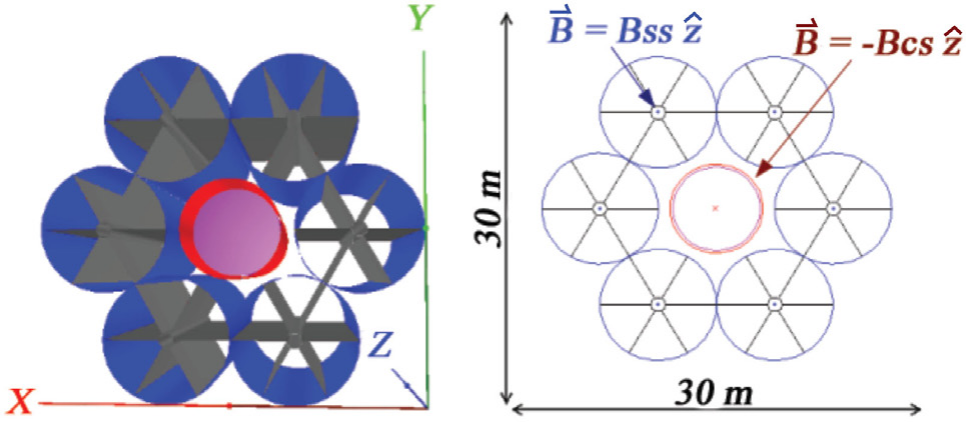
**The NIAC 6 + 1 extendable solenoid shield: shield solenoid coils (blue), carbon-fiber support structures (black), compensation solenoid coil (red), and habitat (magenta)**. The field regions are shown in the *xy* view on the right. The shield solenoid flux density is B_ss_ = 1T. The flux density of the compensation coil B_CS_ is chosen to minimize the net flux density in the habitat.

The magnetic field, coil, and support structures of the 6 + 1 extendable solenoid shield configuration in the simulation are shown in Figure 10. Six, 8-m-diameter, 20-m-long solenoid shield coils surround a 6-m-diameter, 10-m-long, air-filled cylindrical habitat composed of 1.8-cm-thick aluminum. A 6.4-m-diameter, 20 m compensation solenoid coil surrounds the habitat to reduce the magnetic flux density to an acceptable level. In the simulation, a uniform 1 T field is present in the cylindrical volumes delimited by the solenoid shield coil dimensions, elsewhere the field is zero.

The YBCO solenoid shield coils are represented as 111-μm-thick copper cylinders. The support structures are composed of a 1-m-diameter, 1-cm-thick, 20-m-long graphite cylinder located in the center of the coil. The radial spokes are composed of six 2.5-mm-thick, 3.5-m-wide, 20-m-long graphite plates. The compensation solenoid consists of a 111-μm-thick copper cylinder and a 2.4-mm-thick graphite support cylinder. The composition and mass of the structural elements in the simulation, shield and habitat, are reported in Table S1 in Supplementary Material.

The annual BFO dose equivalent for the barrel region acceptance of the 6 + 1 extendable solenoid shield and the 1.8-cm-thick aluminum habitat (NIAC Phase I) are reported in Table 4. A total of 500 M protons and He nuclei, and 330 M, *Z* > 2 nuclei were generated on the surface of the 30 m × 30 m × 30 m cube positioned around the center of the habitat. The shielding performance of the habitat was obtained with 500 M protons and He nuclei, and 150 M, *Z* > 2 nuclei.

**Table 4:** **The dose levels of the 6 + 1 extendable solenoid shield and NIAC habitat**.

Configuration	BL (Tm)	M_sim_ (t)	M_eng_ (t)	BFO (cSv/y)
HTSC 6 + 1 extendable solenoid shield	6.3	46.2	49.5	30.9 ± 0.9
NIAC Phase I habitat	–	10.1	–	36.7 ± 1.1

*The average value of the field integral across the 8-m-diameter cylindrical coil is quoted. The first mass quoted refers to the shield mass present in the Monte Carlo simulation. The second is the mass estimate from the engineering designs. The annual BFO dose equivalents refer to the GCR flux at solar minimum (1) and the barrel region acceptance (Figure 9). The quoted uncertainties represent the root-mean-square deviation of the average dose recorded in the six water cylinders (Figure 5)*.

The 6 + 1 extendable solenoid shield configuration provides an additional ~20% reduction with respect to the habitat dose level. The dose reduction, normalized to the habitat dose, is comparable to the results for the two HTSC toroid configurations of the ESA study (Table 2).

### 5.1 Dose Evaluation over the Full Acceptance

**Figure 11:**
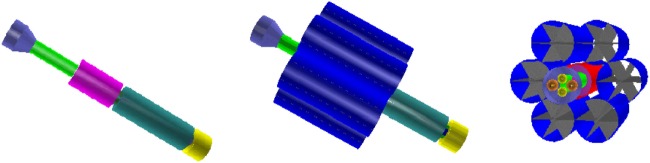
**The NEA chemical propulsion spacecraft in the simulation: re-entry vehicle (violet), access tube (light green), habitat (magenta), liquid hydrogen container (dark green), liquid oxygen container (yellow), YBCO shield solenoid coils (blue), YBCO compensation solenoid coil (red), and carbon fiber support structures of the solenoid coils (gray)**. The liquid methane (orange) and liquid oxygen (yellow) containers of the re-entry vehicle are visible in the view on the right.

The 6 + 1 solenoid shield was employed in a preliminary spacecraft design for a mission to a near Earth asteroid (NEA). The additional spacecraft elements have been included in the simulation (Figure 11) to evaluate their influence on the full acceptance dose.

One side of the habitat is connected to a chemical propulsion system consisting of liquid hydrogen and oxygen tanks, and a combustion chamber. On the other side, an access tube connects the habitat to a re-entry vehicle containing liquid methane and oxygen tanks. The spherical and cylindrical fuel tanks, combustion chamber, access tube, re-entry vehicle, and hatches are composed of 1.8-cm-thick aluminum. The total spacecraft mass in the simulation, including the 6 + 1 extendable solenoid shield and habitat (Table S1 in Supplementary Material), is 88 t. A roughly equivalent mass, 19 and 22 t, has been added on each side of the habitat.

The composition and mass of the additional spacecraft structures are listed in Table S2 in Supplementary Material. The dose evaluation was made without the liquid propellants of the spacecraft propulsion system in order to reduce the computation time.

The annual GCR dose equivalents of the NEA spacecraft and 6 + 1 extendable solenoid shield configurations are compared in Tables 5 and 6 for the barrel and endcap region acceptances, respectively. The dose received from the GCR of the barrel region increases due to the presence of the additional structures of the spacecraft. A decrease in the doses due to the GCR generated in the endcap regions is observed. The net effect of the additional passive shielding elements is negligible on the full acceptance dose.

**Table 5:** **Annual GCR dose equivalents (cSv/y) at solar minimum, in the barrel region (Figure 9) for the 8 Tm extendable solenoid shield and the NEA chemical propulsion spacecraft configurations**.

	6 + 1 Shield			6 + 1 Shield and Spacecraft		
Z	Skin	BFO	Body	Skin	BFO	Body
1	7.9 ± 0.3	7.6 ± 0.3	7.4 ± 0.2	9.5 ± 0.3	9.1 ± 0.5	8.8 ± 0.2
2	3.8 ± 0.2	3.4 ± 0.3	3.4 ± 0.1	4.4 ± 0.3	4.1 ± 0.7	3.8 ± 0.1
3–10	15.4 ± 0.7	10.8 ± 0.5	5.2 ± 0.3	15.6 ± 7.9	11.1 ± 5.5	5.4 ± 2.8
11–20	12.7 ± 0.8	6.9 ± 0.5	5.2 ± 0.4	12.9 ± 7.1	6.7 ± 3.8	5.5 ± 3.1
21–28	5.7 ± 0.8	2.2 ± 0.3	2.0 ± 0.3	5.9 ± 3.1	2.2 ± 1.1	2.1 ± 1.2
Total	45.5 ± 1.4	30.9 ± 0.9	23.2 ± 0.6	48.3 ± 11.1	33.2 ± 6.8	25.6 ± 4.4
Fraction of 6 + 1 Shield dose				1.06 ± 0.25	1.07 ± 0.22	1.10 ± 0.19

*The quoted uncertainties represent the root-mean-square deviation of the average dose recorded in the six water cylinders (Figure 5)*.

**Table 6:** **Annual GCR dose equivalents (cSv/y) at solar minimum, in the endcap regions (Figure 9) for the 8 Tm extendable solenoid shield and the NEA chemical propulsion spacecraft configurations**.

	6 + 1 Shield			6 + 1 Shield and Spacecraft		
Z	Skin	BFO	Body	Skin	BFO	Body
1	6.5 ± 0.4	6.1 ± 0.5	6.0 ± 0.2	5.9 ± 0.1	5.5 ± 0.2	5.4 ± 0.1
2	2.8 ± 0.3	2.3 ± 0.5	2.2 ± 0.1	2.3 ± 0.3	1.9 ± 0.2	1.9 ± 0.1
3–10	7.1 ± 1.3	3.5 ± 0.7	2.3 ± 0.4	5.4 ± 2.1	2.9 ± 1.2	1.8 ± 0.5
11–20	5.7 ± 1.2	1.9 ± 0.5	1.9 ± 0.4	4.1 ± 2.1	1.7 ± 0.9	1.4 ± 0.6
21–28	2.4 ± 0.8	0.4 ± 0.2	0.7 ± 0.2	1.8 ± 0.7	0.4 ± 0.2	0.5 ± 0.2
Total	24.5 ± 2.0	14.2 ± 1.1	13.1 ± 0.6	19.5 ± 3.1	12.4 ± 1.5	11.0 ± 0.8
Fraction of 6 + 1 Shield dose				0.80 ± 0.14	0.87 ± 0.12	0.84 ± 0.07

*The quoted uncertainties represent the root-mean-square deviation of the average dose recorded in the six water cylinders (Figure 5)*.

The ratio of the full acceptance, NEA spacecraft skin, BFO, and body doses to the corresponding 6 + 1 shield doses are respectively 0.97 ± 0.22, 1.01 ± 0.19, and 1.01 ± 0.16. A 50 m × 50 m × 50 m generation cube was used for the NEA spacecraft dose estimation. The NEA spacecraft results are based on 700 M protons and He nuclei, and 365 M, *Z* > 2 nuclei.

The most significant dose increase in Table 5 is observed for the GCR protons due to the increase of secondaries created in the extended spacecraft structures. The charged secondaries, produced by interaction of the GCR in the structures aligned along the cylindrical axis of the spacecraft, enter the field volume and are deflected toward the habitat. The effect is illustrated in Figure S1 in Supplementary Material. In order to be effective, the passive shielding elements of the spacecraft should be placed to obstruct the passage of particles arriving at both ends of the cylindrical volumes of the habitat and solenoid coils.

### 5.2 The 6 + 1 Extendable Solenoid Shield Performance

The effectiveness of the magnetic shield is defined by the comparison of the dose levels with and without the field, which indicates explicitly the contribution of the field to the overall dose reduction. The evaluation was performed for the 6 + 1 extendable solenoid shield of Figure 10, and the larger mass habitat of the NIAC Phase II study, which includes water and food volumes. The NIAC Phase II habitat is shown in Figure S2 in Supplementary Material. The dimensions of the aluminum habitat and water volume, and the composition and dimensions of the food volume are listed in Table S3 in Supplementary Material.

The annual GCR dose equivalents of the two habitats are compared in Table S4 in Supplementary Material. The increase in thickness of the aluminum wall from 1.8 to 4 cm reduces the dose levels by ~15% for the factor 3.5 increase in the habitat mass.

The dose levels of the 6 + 1 extendable solenoid shield, with and without the magnetic field, are presented in Table 7. The reduction due to the 6.3 Tm field represents ~5% of the total dose reduction. A total of 500 M protons and He nuclei, and 205 M, *Z* > 2 nuclei were generated to produce the field off dose estimates, the corresponding numbers for the field on estimates are 500 and 330 M, respectively.

**Table 7:** **The Geant3 annual GCR dose equivalents (cSv/y) at solar minimum for the 6 + 1 solenoid configuration, with and without the 6.3 Tm field for the acceptance corresponding to the barrel region (Figure 9)**.

	6 + 1 no field			6 + 1 field		
Z	Skin	BFO	Body	Skin	BFO	Body
1	10.4 ± 0.4	9.6 ± 0.4	9.4 ± 0.3	9.5 ± 0.6	8.5 ± 0.4	8.5 ± 0.3
2	4.9 ± 0.4	4.2 ± 0.2	4.0 ± 0.1	4.3 ± 0.2	3.8 ± 0.1	3.8 ± 0.1
3–10	11.5 ± 1.0	8.0 ± 0.9	4.1 ± 0.4	11.6 ± 1.1	8.2 ± 0.8	4.0 ± 0.4
11–20	7.9 ± 1.1	4.5 ± 0.7	3.4 ± 0.5	8.2 ± 1.2	4.5 ± 0.3	3.4 ± 0.4
21–28	2.8 ± 0.7	1.1 ± 0.3	1.0 ± 0.2	2.9 ± 0.7	1.1 ± 0.3	1.1 ± 0.3
Total	37.5 ± 1.7	27.6 ± 1.3	22.0 ± 0.7	36.5 ± 1.9	26.1 ± 1.0	20.8 ± 0.7
Fraction field on/off				0.97 ± 0.07	0.95 ± 0.06	0.95 ± 0.04

*The quoted uncertainties represent the root-mean-square deviation of the average dose recorded in the six water cylinders (Figure 5)*.

### 5.3 Geant3/Geant4 Comparison

The Geant3 simulation used for the radiation studies is compiled as a C++ program. The Geant3 user interface routines are prototyped in C++ with CFORTRAN (17). The routines are called from the C++ code to generate the GCR spectra, describe the materials and geometry of the spacecraft and magnetic shield configurations, and record the ionization losses in the water cylinders. The same C++ routines were used with Geant4. A comparison of the dose estimates of the two simulations, using the same methodology for the dose evaluation, indicates the influence of the different physics models implemented in the two simulations.

The Geant3 and Geant4 GCR free space dose equivalents at solar minimum are compared in Table 8. The total dose levels in the 2-mm-thick layers, skin and BFO, are 35 and 30% lower in Geant4, whereas the total dose recorded in the water cylinder (body) agree. The Geant4 proton dose equivalents are 10–15% higher. The Geant4 dose equivalents for the *Z* ≥ 2 nuclei are lower, except for the BFO and body doses of the *Z* > 20 nuclei.

**Table 8:** **Annual GCR dose equivalents (cSv/y) in free space at the solar minimum**.

	Geant3			Geant4		
Z	Skin	BFO	Body	Skin	BFO	Body
1	10.8	11.3	11.1	12.2	12.9	12.8
2	5.3	5.2	5.1	5.8	4.6	4.8
3–10	35.9	22.2	11.8	13.6	6.1	7.4
11–20	38.4	16.6	14.8	19.9	9.2	11.4
21–28	27.3	7.1	8.7	24.7	11.1	14.3
Total	117.7	62.4	51.5	76.2	43.9	50.7

*The effect of the generation statistics on the quoted free space doses is negligible (~1%)*.

QGSP-BERT-HP and QBBC are Geant4 physics lists that group preselected models for hadron physics. QGSP-BERT-HP contains the quark-gluon string precompound model, coupled with the Bertini cascade model for proton and neutrons below 10 GeV (18). QBBC is a physics list containing a combination of various models created for space applications, radiation biology, and radiation protection (19). The Geant4 results were obtained with 50 M protons and He nuclei (QGSP-BERT-HP), 5 M, *Z* > 2 nuclei (QGSP-BERT-HP), and 5 M, *Z* > 2 (QBBC). No significant difference was observed in the Geant4 free space doses generated with the two different physics lists.

The Geant4 dose estimates for the 6 + 1 extendable solenoid shield are reported in Table 9. The contribution of the 6.3 Tm field to the total dose reduction is ~10%. The Geant4 field on and off results were obtained with 50 M protons and He nuclei, and 50 M, *Z* > 2 nuclei. The QGSP-BERT-HP was used for the hadron and nuclear interactions.

**Table 9:** **The Geant4 annual GCR dose equivalents (cSv/y) at solar minimum for the 6 + 1 solenoid configuration (NIAC Phase I habitat), with and without the 6.3 Tm field for the acceptance corresponding to the barrel region (Figure 9)**.

	6 + 1 no field			6 + 1 field		
Z	Skin	BFO	Body	Skin	BFO	Body
1	10.2 ± 0.6	9.3 ± 0.8	9.8 ± 0.4	9.1 ± 0.8	8.4 ± 0.4	8.3 ± 0.3
2	3.7 ± 0.7	3.2 ± 0.3	3.2 ± 0.1	3.0 ± 0.2	2.6 ± 0.7	3.0 ± 0.2
3–10	5.1 ± 0.4	3.3 ± 0.4	3.7 ± 0.2	3.9 ± 0.2	2.9 ± 0.3	3.3 ± 0.2
11–20	8.0 ± 0.7	4.5 ± 0.6	5.4 ± 0.4	7.4 ± 0.9	4.1 ± 0.6	5.0 ± 0.4
21–28	9.4 ± 0.8	5.3 ± 0.6	6.7 ± 0.4	9.8 ± 1.1	5.6 ± 0.9	6.6 ± 0.5
Total	36.4 ± 1.5	25.6 ± 1.3	28.8 ± 0.7	33.2 ± 1.7	23.6 ± 1.4	26.2 ± 0.8
Fraction field on/off				0.91 ± 0.06	0.92 ± 0.07	0.91 ± 0.04

*The quoted uncertainties represent the root-mean-square deviation of the average dose recorded in the six water cylinders (Figure 5)*.

The relative performance of passive shielding in the two simulations may be compared using the Geant3 results for the NIAC Phase I habitat (Table S4 in Supplementary Material) and free space (Table 8). In the Geant3 simulation, the skin, BFO, and body dose equivalents of the NIAC Phase I habitat represent, respectively, 33.6 ± 1.8%, 22.3 ± 2.4%, and 24.7 ± 1.8% of the annual free space doses. The corresponding Geant4, NIAC Phase I habitat dose equivalents, 50.2 ± 2.8, 37.2 ± 2.3, and 39.5 ± 1.3 cSv/y, represent 34.2 ± 3.7%, 15.3 ± 5.2%, and 23.3 ± 2.6% of the corresponding free space doses in Table 8. The results for the two simulations indicate a comparable dose reduction for the 1.8-cm-thick aluminum habitat. The Geant4, NIAC Phase I habitat dose estimates were obtained with 25 M protons and He nuclei, and 23.5 M, *Z* > 2 nuclei.

In contrast to the Geant3 results in Table 7, a dose reduction in the presence of the field is observed in the Geant4 results for *Z* > 2 GCR nuclei, which results in a larger contribution of the field to the overall dose reduction. The difference is explained by the significantly thicker NIAC Phase II habitat used for the Geant3 performance evaluation, which enhances the performance of the passive shielding element for *Z* > 2 GCR nuclei (Table S4 in Supplementary Material).

The 1.8-cm-thick aluminum NIAC Phase I habitat was used for the Geant4 performance evaluation in order to compare the results with the continuous-coil toroid shield, and 1.5-cm-thick aluminum habitat, of the SR2S study.

### 5.4 Dose Equivalent and Absorbed Dose

The Geant3 and Geant4, free space absorbed doses are reported in Table 10. The Geant4 absorbed doses are systematically lower for the *Z* ≥ 2 nuclei. The differences between the total skin, BFO, and body absorbed dose are 25, 12, and 5%, respectively.

**Table 10:** **Annual GCR absorbed doses (cGy/y) in free space at solar minimum**.

	Geant3			Geant4		
Z	Skin	BFO	Body	Skin	BFO	Body
1	8.3	8.4	8.4	8.3	8.7	8.6
2	3.6	3.2	3.3	3.2	2.8	2.8
3–10	3.0	1.9	1.7	2.1	1.4	1.6
11–20	2.7	1.3	1.0	1.3	0.7	0.8
21–28	4.1	1.3	0.9	1.3	0.6	0.7
Total	21.7	16.1	15.3	16.2	14.2	14.5

*The effect of the generation statistics on the quoted free space doses is negligible (~1%)*.

The free space absorbed doses are dominated by protons and He nuclei, the dominant components of GCR (~95%). The dose equivalent is obtained by multiplying the absorbed dose by the quality factor (equation (4)). Due to the strong dependence of the ionization loss on charge (Equation (1)), which affects the weighting of the quality factor, the GCR free space dose equivalents are dominated by the contribution of the *Z* > 2 nuclei (Table 8).

The estimated contribution of the field to the overall reduction of the absorbed dose for the 6 + 1 extendable solenoid shield is reported in Tables 11 and 12. The results are comparable to those obtained for the dose equivalents, i.e., ~5% with Geant3 (Table 7) and ~10% with Geant4 (Table 9).

**Table 11:** **The Geant3 annual GCR absorbed dose (cGy/y) at solar minimum for the 6 + 1 solenoid configuration, with and without the 6.3 Tm field for the acceptance corresponding to the barrel region (Figure 9)**.

	6 + 1 no field			6 + 1 field		
Z	Skin	BFO	Body	Skin	BFO	Body
1	7.19 ± 0.25	6.74 ± 0.17	6.81 ± 0.18	6.53 ± 0.28	6.07 ± 0.18	6.15 ± 0.21
2	2.73 ± 0.06	2.42 ± 0.07	2.46 ± 0.04	2.67 ± 0.07	2.39 ± 0.03	2.47 ± 0.04
3–10	1.22 ± 0.05	0.93 ± 0.04	0.83 ± 0.03	1.26 ± 0.06	0.98 ± 0.04	0.86 ± 0.03
11–20	0.68 ± 0.06	0.43 ± 0.06	0.33 ± 0.02	0.72 ± 0.09	0.43 ± 0.02	0.35 ± 0.02
21–28	0.46 ± 0.09	0.21 ± 0.07	0.17 ± 0.02	0.48 ± 0.10	0.20 ± 0.05	0.19 ± 0.04
Total	12.28 ± 0.28	10.73 ± 0.21	10.60 ± 0.19	11.66 ± 0.32	10.07 ± 0.19	10.02 ± 0.22
Fraction field on/off				0.95 ± 0.03	0.94 ± 0.03	0.95 ± 0.03

*The quoted uncertainties represent the root-mean-square deviation of the average dose recorded in the six water cylinders (Figure 5)*.

**Table 12:** **The Geant4 annual GCR absorbed dose (cGy/y) at solar minimum for the 6 + 1 solenoid configuration (NIAC Phase I habitat), with and without the 6.3 Tm field for the acceptance corresponding to the barrel region (Figure 9)**.

	6 + 1 no field			6 + 1 field		
Z	Skin	BFO	Body	Skin	BFO	Body
1	6.68 ± 0.20	6.39 ± 0.30	6.43 ± 0.21	5.70 ± 0.23	5.39 ± 0.13	5.42 ± 0.14
2	2.13 ± 0.17	1.89 ± 0.11	1.93 ± 0.07	1.92 ± 0.09	1.74 ± 0.16	1.82 ± 0.08
3–10	1.13 ± 0.03	0.86 ± 0.03	0.92 ± 0.02	1.04 ± 0.03	0.83 ± 0.03	0.89 ± 0.02
11–20	0.61 ± 0.03	0.40 ± 0.03	0.45 ± 0.02	0.59 ± 0.05	0.39 ± 0.03	0.45 ± 0.02
21–28	0.49 ± 0.05	0.30 ± 0.03	0.36 ± 0.02	0.52 ± 0.05	0.31 ± 0.04	0.36 ± 0.02
Total	11.04 ± 0.27	9.84 ± 0.32	10.09 ± 0.22	9.77 ± 0.26	8.66 ± 0.21	8.94 ± 0.16
Fraction field on/off				0.88 ± 0.03	0.88 ± 0.04	0.89 ± 0.03

*The quoted uncertainties represent the root-mean-square deviation of the average dose recorded in the six water cylinders (Figure 5)*.

The result of the comparative performance evaluation does not depend on the dose quoted. It would seem more appropriate to quote the dose equivalent, which better reflects the higher radiation risk associated with the larger energy losses of high charge nuclei (20).

## 6 SR2S Results

The HTSC racetrack coils of the continuous-coil toroid shield (Figure 4) are described in the simulation by a cable core with a density of 3.0g/cm^3^, composed of 57.4% aluminum, 8.6% MgB_2_, 23% titanium, and 11% SiO_2_. The cables are surrounded by 1.2-cm-thick aluminum.

A 0.6-mm-thick KEVLAR sheath (90 kg) surrounds each coil. The total mass of the 120 coil toroid is 79 t. The toroid is supported by a 5.5-m-diameter, 4.4-cm-thick, 8.6-m-long Al-B4C cylinder shell (60% boron), with a density of 2.6 g/cm^3^, and a mass of 16.8 t. The total mass of the 8 Tm field configuration is 95.8 t. The individual contributions of the different elements to the total mass are shown in Figure S3 in Supplementary Material.

**Figure 12:**
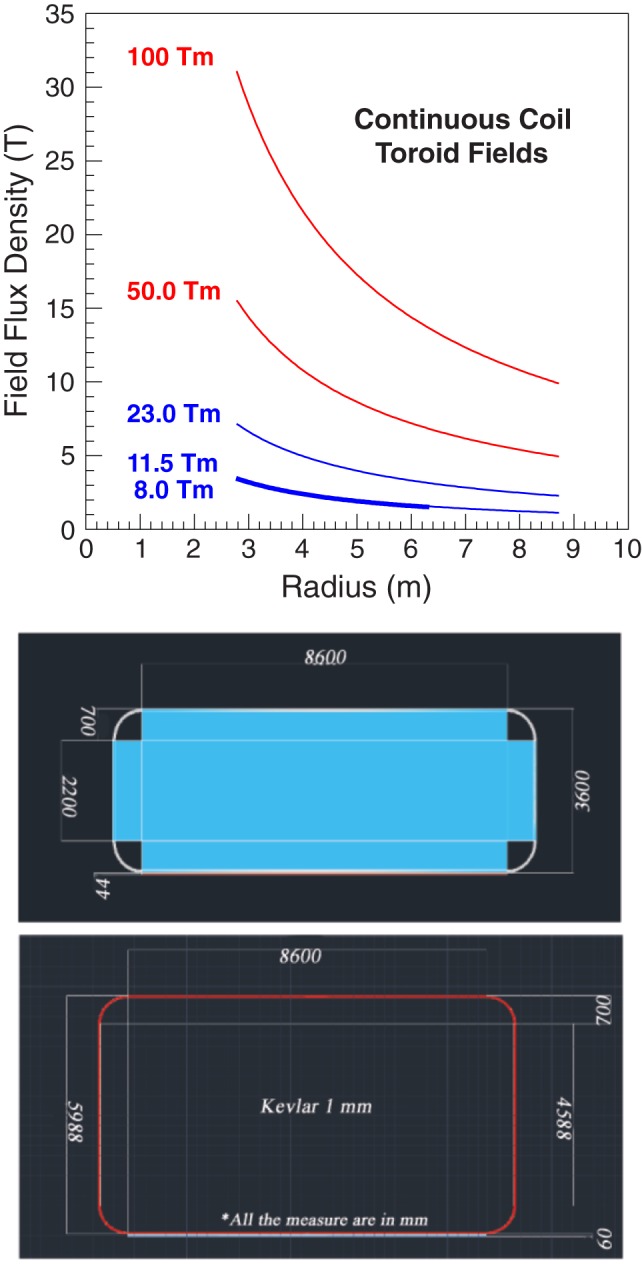
**(Top Panel)** The toroid fields used for the performance evaluation. The field flux densities of 50 and 100 Tm require current densities that exceed the performance of present-day HTCS. **(Bottom Panel)** The dimensions (millimeter) of the racetrack coils of the 8 Tm (top) and 11.5 (23) Tm (bottom) continuous-coil toroid shield configurations.

The shield performance with GCR protons and *Z* ≥ 2 nuclei was evaluated with the field integral of 8 Tm. Higher field values were used to study the evolution of the proton and He nuclei doses with field strength and shield mass, and in particular the contributions of the charged secondaries and neutrons. The toroid fields used in the simulation are shown in Figure 12.

The 11.5 Tm shield configuration is obtained by increasing the coils dimensions. With the larger dimension coil, and an increase of the current density to the limiting value of the cable dimensions, the integral flux attains a value of 23 Tm. Figure 12 shows the coil dimensions of the 8 Tm, and 11.5 (23) Tm configurations. The total shield mass in the simulation of the larger dimension coil configurations is 137 t.

The 4.5-m-diameter, 6-m-long Columbus habitat (Figure 4) is composed of a 1.5-cm-thick aluminum cylindrical shell and two 3.0-cm-thick aluminum endcaps. The mass of the habitat in the simulation is 4.36 t.

### 6.1 The Continuous-Coil Toroid Shield Performance

The annual GCR dose equivalents of the continuous-coil toroid shield, with and without the 8 Tm field, are compared in Table 13. The dose levels refer to the dose received in the barrel region of the 30 m × 30 m × 30 m cube, positioned around the center of the Columbus habitat. The Geant4 QBBC physics list was used for the hadron and nuclear interactions.

**Table 13:** **The Geant4 annual GCR dose equivalents (cSv/y) for the continuous-coil toroid (CCT) shield configuration, with and without the 8 Tm field, for the acceptance corresponding to the barrel region (Figure 9)**.

	CCT no field			CCT field		
Z	Skin	BFO	Body	Skin	BFO	Body
1	16.3 ± 0.5	15.2 ± 0.9	15.1 ± 0.3	12.3 ± 0.9	11.3 ± 0.4	11.6 ± 0.3
2	3.94 ± 0.13	3.84 ± 0.36	3.88 ± 0.08	3.56 ± 0.43	3.10 ± 0.27	3.40 ± 0.07
3–10	1.52 ± 0.08	1.32 ± 0.12	1.38 ± 0.08	1.17 ± 0.07	1.03 ± 0.04	1.06 ± 0.04
11–20	1.01 ± 0.10	0.80 ± 0.10	0.84 ± 0.09	0.75 ± 0.06	0.60 ± 0.04	0.65 ± 0.04
21–28	1.04 ± 0.13	0.74 ± 0.12	0.84 ± 0.14	0.90 ± 0.08	0.64 ± 0.11	0.72 ± 0.09
Total	23.8 ± 0.5	21.9 ± 1.0	22.1 ± 0.4	18.7 ± 1.0	16.7 ± 0.5	17.4 ± 0.3
Fraction field on/off				0.79 ± 0.06	0.76 ± 0.05	0.79 ± 0.02

*The quoted uncertainties represent the root-mean-square deviation of the average dose recorded in the six water cylinders (Figure 5)*.

The magnetic field produces an additional 20–25% reduction of the dose levels provided by the passive shielding of the shield and habitat material. The corresponding annual skin, BFO, and body dose equivalents of the Columbus habitat are, respectively, 35.6 ± 0.9, 28.1 ± 1.3, and 30.2 ± 0.4 cSv/y. The magnetic shield reduces the habitat doses by 40–50%.

**Figure 13:**
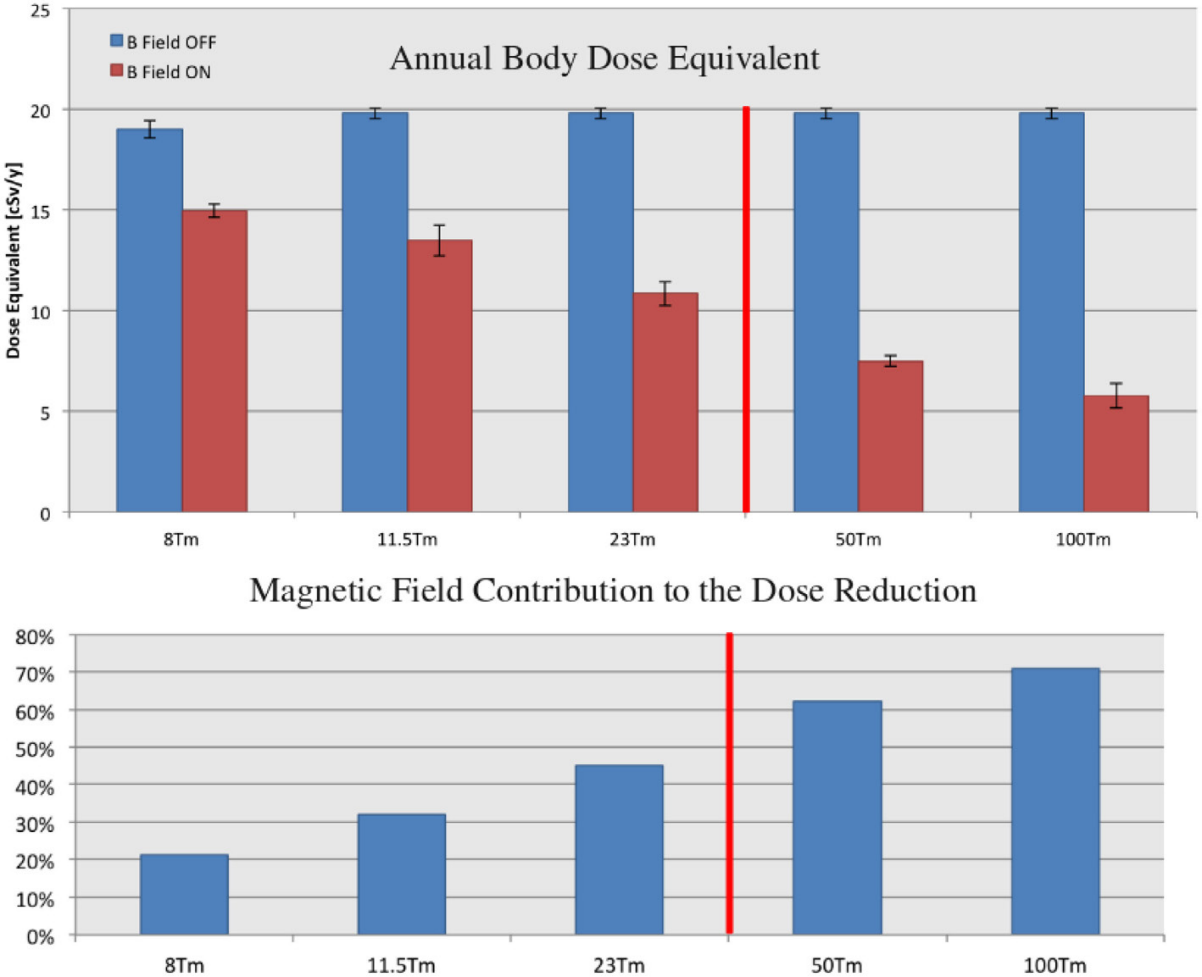
**The annual body dose equivalent doses due to GCR protons and He nuclei, with the field off and on, for the 8, 11.5, 23, 50, and 100 Tm continuous-coil toroids (top), and the corresponding contributions of the field to the dose reduction (bottom)**. The 50 and 100 Tm configurations require field flux densities that exceed the performance of present-day HTSC. The quoted doses refer to the barrel region acceptance (Figure 9). The displayed errors represent the root-mean-square deviation of the average dose recorded in the six water cylinders (Figure 5).

The evolution of the dose reduction with field strength and shield mass is illustrated in Figure 13. The reported annual body dose equivalents refer to GCR proton and He nuclei, which represent the dominant contribution (~85%) to the estimated total doses (Table 13). The 50 and 100 Tm fields, which require current densities exceeding the performance of present-day HTSC, provide an indication of the dose reduction expected for significantly higher field flux densities.

The required increase in the screen mass, for the larger integral field values, results in a higher field off dose level due to an increase in the secondary dose. The increase is more than compensated by the magnetic field, which results in a steady decrease of the total dose with increasing field strength. The contribution of the field to the overall dose reduction is 45% at 23 Tm, the technological limit of the present-day HTSC. At 100 Tm, the field represents 70% of the observed dose reduction.

In general, an increase in material thickness is accompanied by an increase in the secondary dose for the GCR protons and He nuclei. For integral field values BL ≤ 8 Tm, the proton dose levels of the shielding configurations studied exceed the free space dose level. Above 11.5 Tm, the combination of mass and field of the continuous-coil toroid configurations result in proton dose levels below the free space value.

### 6.2 Secondary Particle Production

**Figure 14:**
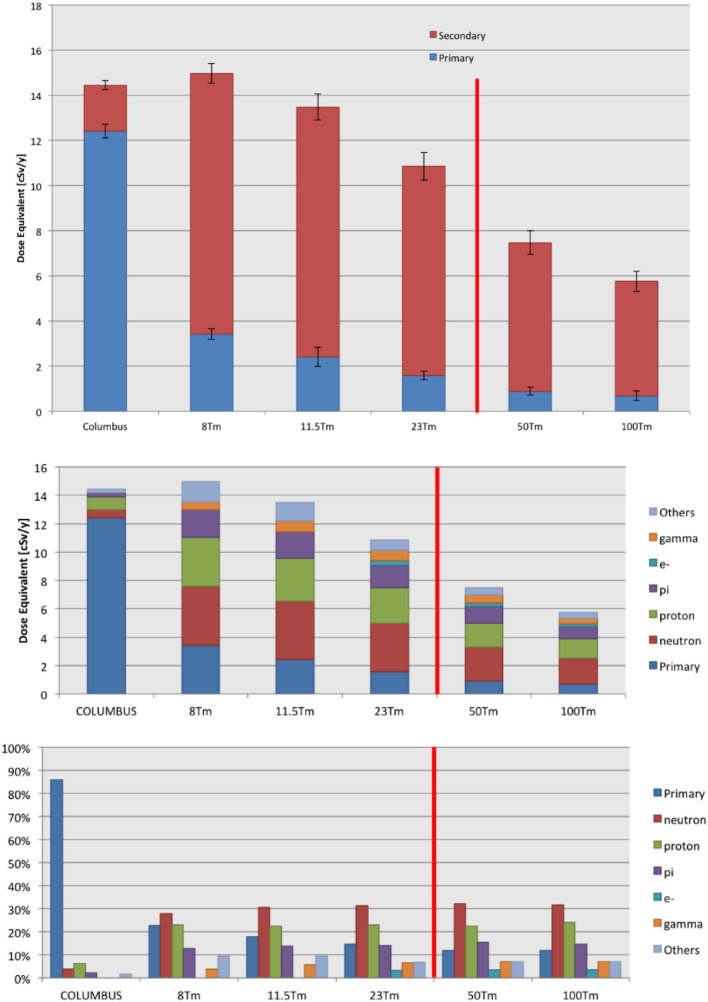
**(Top panel)** The contributions of the primary GCR proton and He nuclei, and the secondaries produced in the material of the habitat and magnet shield to the annual body dose equivalent. The error bars represent the root-mean-square deviation of the average primary and secondary doses recorded in the six water cylinders (Figure 5). The quoted doses refer to the barrel region acceptance (Figure 9). The 50 and 100 Tm configurations require field flux densities that exceed the performance of present-day HTSC. **(Bottom panel)** The annual body dose equivalent of the primary GCR proton and He nuclei, and the secondary particles (top), and the contribution of each category to the total dose (bottom). The 50 and 100 Tm configurations require field flux densities that exceed the performance of present-day HTSC.

The effect of the magnetic field on the dose due to the secondary particles was studied in detail in the SR2S study. Figure 14 shows the contributions of the primary GCR protons and He nuclei, and their secondaries, for the Columbus habitat alone, and the five field configurations.

The presence of the 8 Tm field does not compensate the dose due to the secondaries created in the shield material; consequently, the total dose due to protons and He nuclei exceed the level of the Columbus habitat. A decrease of both the primary and secondary doses is observed as the field integral BL increases.

The individual contributions of the primary and secondary particles are shown in Figure 14. The principal contributions to the secondary dose are due to protons and neutrons. The reported neutron dose levels refer to the ionization losses recorded in the water cylinders of the secondary particles created by neutron interactions in the material of the shield and habitat. The individual contributions of the secondaries denoted “others” include the mesons and baryons not explicitly quoted, and light nuclei.

**Figure 15:**
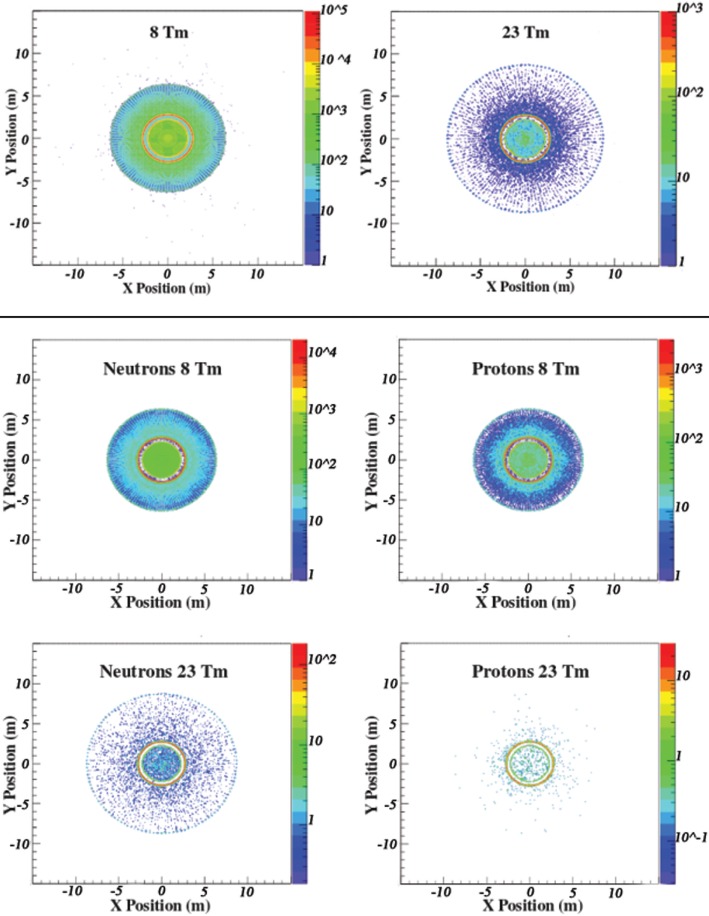
**(Top panel)** The vertex distributions of the generated secondaries that contribute to the dose in the 8 and 23 Tm continuous-coil toroid shield configurations. The largest contribution to the secondary dose is due to interactions in the Al-B4C support cylinder surrounding the habitat, denoted by the red circular regions located inside the field volume in the *xy* projections. **(Middle and Bottom panels)** The vertex distributions of the generated secondary neutrons and protons that contribute to the dose in the 8 and 23 Tm continuous-coil toroid shield configurations.

The vertex distributions of the secondaries created by the primary GCR protons and He nuclei in the materials of the magnet shield and habitat, which contribute to the dose in the 8 and 23 Tm continuous-coil toroid shield configurations, are shown in top panel of Figure 15. The particles created in Al-B4C support cylinder surrounding the habitat are the principal source of the secondary dose.

The geometric acceptance limits the contribution of the particles created at a greater distance from the habitat. The contribution of the charged secondaries is further reduced by the presence of the magnetic field. The larger dimension coils and higher BL are responsible for the difference in the two vertex distributions.

The vertex distributions of the secondary neutrons and protons that contribute to the recorded dose levels are presented in the bottom panel of Figure 15. The effect of the field on the secondary protons results in a shorter radial extension of the volume, which contributes to the dose, compared to the region corresponding to the neutron contribution.

## 7 Discussion

### 7.1 Physics Models and Dose Estimation

The Geant3 free space BFO dose equivalents of the ESA study (Table 1) were compared to previous estimates (16) to verify the dose calculation. The comparison of the Geant3 and Geant4 free space doses provides an estimate of the effect of the different physics models on the dose determination. A significant difference is observed in the dose levels due to GCR nuclei in the two simulations.

In Geant3, the interactions of the *Z* > 2 nuclei are dominated by ionization loss. In Geant4, the situation is modified by the presence of inelastic nuclear interactions that increases the level of secondary particle production. The difference is explained by the more extensive hadron interaction models available in Geant4.

The different weighting between ionization and the hadron interactions in the two simulations is illustrated by the systematically lower Geant4 free space doses for the *Z* > 2 nuclei in Tables 8 and 10. The lower rate of ionization of the primary GCR nuclei compared to secondary productions implies a relatively lower shielding efficiency of the material present in the shield structures and spacecraft, analogous to behavior observed for protons, due to the non-contained secondary particles.

The up-to-date hadron models in Geant4 result in ~30% lower estimate for the free space BFO dose equivalent (16). The change in the dose level due to the physics models is significant, on the order of the expected variations due to solar activity (40%) and the contributions of the magnetic field to the estimated dose reductions (10–50%). The Geant4 free space skin doses, 76.2 and 16.2 cGy/y, are in better agreement with the corresponding dose estimates based on the *in situ* measurements of the Mars Science Laboratory, respectively, 67.2 ± 12.0 and 17.6 ± 2.9 cGy/y (21).

The quoted free space dose values allow a comparison of the methodology and underlying physics used to determine the dose. However, they are not a realistic reference to define the efficiency of the shielding configuration, since the effect of the material that must be present (spacecraft) is ignored.

### 7.2 Active and Passive Shielding Elements

In the ESA study, the spacecraft consisted of a habitat and propulsion system. A characteristic of the axial field of the toroid shield is the asymmetric deflection for particles entering the field volume in the two directions parallel to the toroid axis (Figure 8). The material of the propulsion system is present to stop the particles that would be deflected in the direction of the habitat. The spacecraft was used as a reference for the comparison presented in Table 3, which indicates the relative contributions of the passive and active shielding elements to the dose reduction.

The magnet designs of the engineering studies were incorporated in the shield configuration description in the simulations used to provide the dose estimates. The emphasis was placed on the details of the shield design in terms of material composition and location, in order to accurately evaluate the shielding performance of the passive and active elements.

The magnetic shields were not integrated in an overall spacecraft design in the ESA and SR2S studies. The performance evaluations were made with respect to the dose levels of the habitat, limited to the acceptance shielded by the magnetic field volume, the barrel region defined in Figure 9.

### 7.3 Spacecraft Design and Shield Optimization

The 6 + 1 extendable solenoid shield of the NIAC study was used in a preliminary spacecraft design (Figure 11) to extend the dose estimate over the full acceptance. The effect of the additional spacecraft structures was negligible. The dose reduction due to the material aligned along the axis of the cylindrical spacecraft was balanced by the dose increase due to charged secondaries created in structures placed on each side of the habitat (Figure S1 in Supplementary Material).

A further optimization of the overall performance requires a sufficient quantity of material to shield the magnetic field volume and limit the entry of particles that would be deflected in the direction of the habitat. The NIAC study demonstrates the need to integrate an active magnetic shield early in the spacecraft design.

The interplay between the magnetic field, and the passive shielding of the shield and spacecraft materials, requires a detailed knowledge of the geometry (Figure 15). The increase of the NIAC habitat thickness from 1.8 to 4.0 cm (Table S4 in Supplementary Material) indicates the sensitivity of the magnetic shield performance to the mass distribution (Tables 7 and 9).

Mass reduction is a constant concern in each phase of a space project. A potential radiation shield is evaluated in terms of shielding performance and required mass. The original interest in active magnetic shielding was motivated by the possibility to reduce the mass required by a passive shield of equivalent performance. The theme motivated the decision of the NIAC study to abandon the toroidal field of the double-helix solenoid of the earlier ESA study, in favor of an extendable solenoid coil with a lower mass, flexible coil, which results in a larger field integral BL by maximizing *L*.

### 7.4 Performance Comparison of the Advanced Designs

The performance of the NIAC 6 + 1 extendable solenoid and the SR2S continuous-coil toroid shields are presented in Table 14. The performance is expressed in terms of the contribution of the field to the overall dose reduction of the shield, including the passive shielding elements, and the combined shield plus habitat dose level with respect to the habitat dose level. The GCR annual BFO dose equivalent corresponding to the barrel acceptance is used for the comparison.

**Table 14:** **The performance of the NIAC 6 + 1 extendable solenoid (ES) and the SR2S continuous-coil toroid (CCT) shields**.

			BFO Dose Eq. (cSv/y)			
Shield	BL (Tm)	Mass (t)	Field Off	Field On	% Field	% Habitat
NIAC 6 + 1 ES	6.3	35	25.6 ± 1.3	23.6 ± 1.4	7.8 ± 0.6	12.3 ± 1.1
SR2S CCT	8	96	21.9 ± 1.0	16.7 ± 0.5	23.7 ± 1.3	59.4 ± 3.3

*The reported shield masses refer to the mass in the Geant4 simulation. The GCR annual BFO dose equivalents correspond to the barrel region acceptance (Figure 9). The quoted uncertainties represent the root-mean-square deviation of the average dose recorded in the six water cylinders (Figure 5). “% Field” is the dose reduction due to the field. “% Habitat” is the reduction of the habitat dose due to the magnetic shield*.

The 6 + 1 extendable solenoid shield results in a BFO dose equivalent of 23.6 cSv/y, the field is responsible for 8% of the total dose reduction. The dose level of the continuous-coil toroid configuration, 16.7 cSv/y, is 30% lower. The toroidal field contributes at the level of ~25% to the total dose reduction.

The difference observed between the reductions of the habitat dose levels is explained by the relative shielding efficiency for the *Z* > 2 nuclei, which contribute ~50% to the total dose of the solenoid shield (Table 9) and ~15% to the toroid shield dose (Table 13). The passive shielding of the larger mass continuous-coil toroid effectively eliminates the dose contribution of the higher charge nuclei. In comparison, the difference in thickness of the two habitats has a negligible contribution. The ratio of the NIAC-to-Columbus BFO dose equivalents is 0.96 ± 0.08.

The body dose equivalent due to GCR protons and He nuclei exceed the value observed for the Columbus habitat for the 8 Tm continuous-coil toroid configuration (Figure 14). A further improvement in the performance is obtained by increasing the field integral BL. The effect on the proton and He nuclei annual BFO dose equivalents are presented in Table 15.

**Table 15:** **The GCR proton and He nuclei dose reductions for the 8, 11.5, and 23 Tm continuous-coil toroid (CCT) configurations**.

			Body Dose Eq. (cSv/y)			
Shield	BL (Tm)	Mass (t)	Field Off	Field On	% Field	% Habitat
SR2S CCT	8	96	19.0 ± 1.0	14.4 ± 0.6	24 ± 2	1 ± 4
SR2S CCT	11.5	137	20.3 ± 2.1	12.8 ± 1.8	37 ± 9	12 ± 12
SR2S CCT	23	137	20.3 ± 2.1	10.5 ± 1.2	48 ± 6	28 ± 8

*The reported shield masses refer to the mass in the Geant4 simulation. The annual BFO dose equivalents correspond to the barrel region acceptance (Figure 9). The quoted uncertainties represent the root-mean-square deviation of the average dose recorded in the six water cylinders (Figure 5). “% Field” is the dose reduction due to the field. “% Habitat” is the reduction of the habitat dose due to the magnetic shield*.

The 140 t, 23 Tm continuous-coil toroid shield configuration would result in a BFO dose equivalent of ~10 cSv/y. The contribution of the *Z* > 2 nuclei, 15% for the 8 Tm toroid shield (Table 13), may be considered negligible for the higher field, larger mass configuration. The presence of the magnetic field is responsible for 50% of the total reduction. The dose level, which corresponds to the part of the acceptance protected by the magnetic shield, ~75% of the total, is a factor ~5 smaller than the limit for LEO (16).

The performance for higher field integrals of the NIAC extendable solenoid coil shield concept is presented in Ref. (22). A comparable equivalent dose is quoted for a 19 Tm solenoid shield (*B* = 4 T, *L* = 4.75 m). The contribution of the field to the total dose reduction is not indicated.

## 8 Conclusion

The results obtained in the three studies conducted over the last 5 years^2^ provide a realistic view of the current situation of a technology, which has been proposed for nearly 50 years as a solution for the radiation protection required for interplanetary manned space missions. The shielding performance of the engineering designs was evaluated with detailed 3-dimensional Monte Carlo simulations. The simulations are used extensively for the design of particle detectors for accelerator and astrophysics experiments. The same methodology has been used to design and evaluate the particle shields. In addition to the importance of the physics processes, a detailed description of the materials and the detector, or shield geometry is essential in each application.

The two HTSC candidates were identified and used in solenoid and race-track coil toroid magnet configurations in the initial ESA study. A wide survey of possible shielding configurations was made. The magnetic shield performance in terms of the contribution of the passive and active elements, a novelty, was presented.

The succeeding NIAC and SR2S studies concentrated on a single shield concept, which allowed to further develop the engineering design and improve the performance estimate. The results presented in Table 14 represent the outcome of the efforts of the two complementary studies employing solenoid and toroid shield configurations. The best performance is obtained with the continuous-coil toroid shield based on the magnesium diboride HTSC: a 23 Tm configuration, with a mass of 140 t and a BFO dose equivalent of ~10 cSv/y (Table 15). The field accounts for 50% of the dose reduction.

The BFO dose equivalent, corresponding to 75% of the total acceptance, is a factor ~5 smaller than the current limits for LEO (16). The remaining acceptance should be protected by the passive shielding of the spacecraft. The presence of the active magnetic shield would render redundant a passive shielding shelter for SEP events.

A further improvement in performance requires field strengths exceeding the current densities of present-day HTSC. The situation may be improved by future developments in superconducting technology, or possibly by an innovative magnetic field configuration. An initial concept design, a minimal two-coil configuration was studied by SR2S. The unconfined field configuration, composed of two 18 MA-turn, 10 m × 20 m coils, results in a ~40% reduction of the habitat dose level (23).

More realistic configurations for a spacecraft shield, consisting of three or four, 3-coil toroids surrounding the habit are considered. The unconfined fields reach higher values of BL with magnetic flux densities compatible with the performance of the magnesium diboride HTSC. The reduction of the number of racetrack coils lowers the shield mass and reduces the dose due to secondary particles. The challenge is to obtain a superposition of the multiple toroidal fields, which results in an acceptable field intensity in the sensible regions of the spacecraft, and optimizes the shielding efficiency.

There are no established dose limits for interplanetary space travel. The long-term health risks due to a prolonged exposure to GCR are not well known. The two atomic bombings, irradiation during nuclear accidents, the lunar manned missions, and human activity in LEO do not represent exactly the conditions encountered during an interplanetary voyage.

A major effort has been made to develop a more precise assessment of the risk due to radiation exposure in space (24). Among the principal concerns are the biological effects caused by the high charge GCR nuclei. The observed biological effects require a reevaluation of quality factors and relative biological effectiveness (RBE) values used to compute dose levels, and establish future dose limits for interplanetary missions.

*A priori*, the increased biological risk due to the higher ionization energy loss may be compensated by the good efficiency of the passive shielding for the high charge nuclei. The performance of the SR2S continuous-coil toroid is limited by the contribution of secondaries created in the shield mass. If the reduction of the shield mass is not compensated by a sufficient increase of BL, for example, in the multi-toroid configuration, the dose contribution of the high charge GCR may become the limiting factor.

Risk assessment is affected often by the subjective perception of the danger, an aspect that will likely play a role in the planning of the first interplanetary mission, in particular for radiation protection against long-term health risks. A first manned mission to Mars will largely exceed the worldwide impact of the first landing on the Moon. The challenge is considerable in a world preoccupied by the reduction of costs and risks.

## Author Contributions

FA was co-responsable for the INFN Monte Carlo simulation performance evaluation of the SR2S study. RB was the project director of the ESA and SR2S studies. WB was responsable for the Monte Carlo performance evaluations of the ESA and NIAC studies, and co-responsable for the INFN performance evaluation of the SR2S study.

## Conflict of Interest Statement

The authors declare that the research was conducted in the absence of any commercial or financial relationships that could be construed as a potential conflict of interest.

## Footnotes

^1^Funded by grant agreement FP7-SPACE Ref. No. 313224 of the European Union’s 7th Framework Programme (2007–2013).

^2^ESA (2011), NIAC (2012-2013), and SR2S (2013-2015).
