# Supplementary material for: Evaluation of Superconducting Magnet Shield Configurations for Long Duration Manned Space Missions
Source: Front Oncol. 2016 Jun 8;6:97. doi: 10.3389/fonc.2016.00097 (PMC4896949; doi:10.3389/fonc.2016.00097)
Supplement: Supplementary file 1 [file Presentation_1.pdf]

Evaluation of Superconducting Magnet Shield Configurations for Long Duration Manned Space Missions

Supplementary Material

F. Ambroglini[a], R. Battiston[b], W. J. Burger[c,*]

[a]University of Perugia and INFN-Perugia, Via Pascoli, 06123 Perugia, Italy
[b]University of Trento and TIFPA, Via Sommarive 14, 38123 Trento, Italy
[c]FBK, Via Sommarive 18, and TIFPA, Via Sommarive 14, 38123 Trento, Italy

Abstract

A manned mission to Mars would present an important long term health risk to the crew members due to the prolonged exposure to the ionizing radiation of galactic cosmic-rays. The radiation levels would largely exceed those encountered in the Apollo missions. An increase in the passive shielding provided by the spacecraft implies a significant increase of the mass. The advent of superconducting magnets in the early 1960's was considered an attractive alternative. The technology allows to generate magnetic fields capable to deflect the cosmic-rays in a manner analogous to the reduction of the particle fluxes in the upper atmosphere, due to the Earth's dipole magnetic field.

A series of the three studies have been conducted over the last five years, funded successively by European Space Agency (ESA), the NASA Innovative Advanced Concepts (NIAC) program and the Union European's Seventh Framework Programme (FP7). The shielding configurations studied are based on high temperature superconductors, which eliminate the need to operate with liquid helium. The mass estimates of the coils and supporting structure of the engineering designs are based on the current and expected near-future performance of the superconducting materials. In each case, the shield performance, in terms of dose reduction, is provided by a 3-dimensional Monte Carlo simulation, which treats in detail the electromagnetic and hadronic interactions of the galactic-cosmic rays, and the secondary particles they produce in the materials of the shield and spacecraft. A summary of the results of the studies, representing one of the most detailed and comprehensive efforts made in the field, is presented.

Keywords: long duration manned space missions, active magnetic shielding, radiation protection, Monte Carlo simulation

*corresponding author

1. Figures

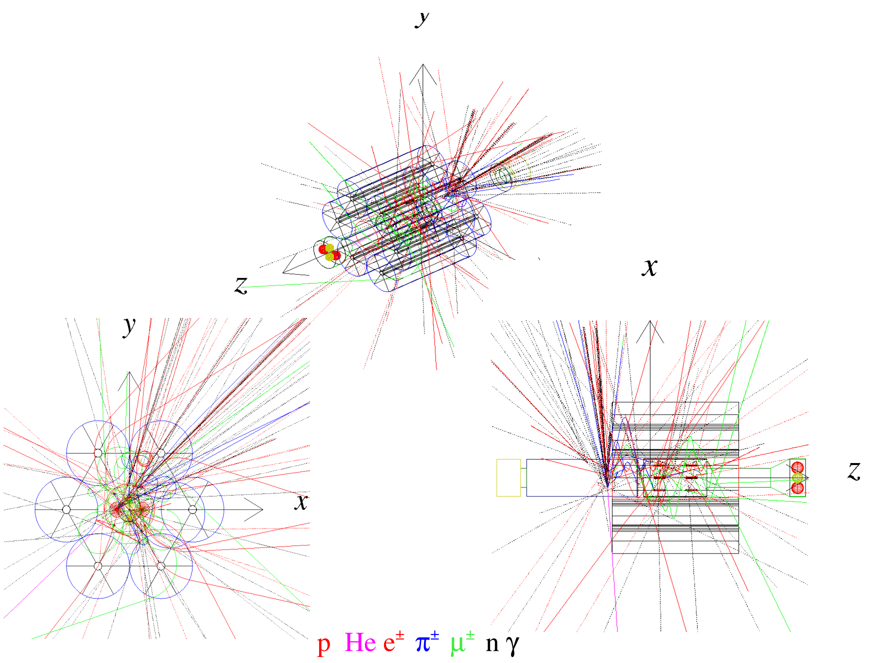

Figure S 1: Simulated events in which a GCR proton or He nuclei generated in the barrel region resulted in an ionization energy loss in the water cylinders. Particle types are indicated by track color. The deflection of primary and secondary particles in the direction of the habitat is visible the *xy* (left) and *xz* (right) projections. The generation of secondaries in the liquid hydrogen container is visible in the *xz* projection (right).

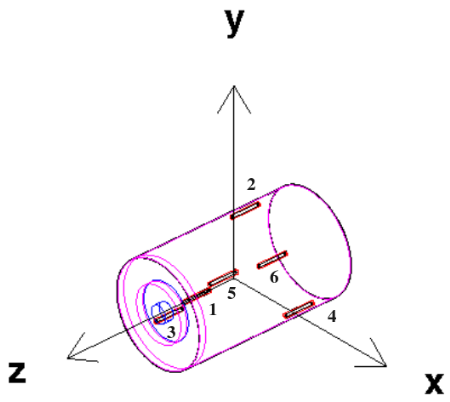

Figure S 2: The NIAC Phase II habitat in the simulation. The food and water annular volumes are placed on the $+\hat{z}$ end of the habitat.

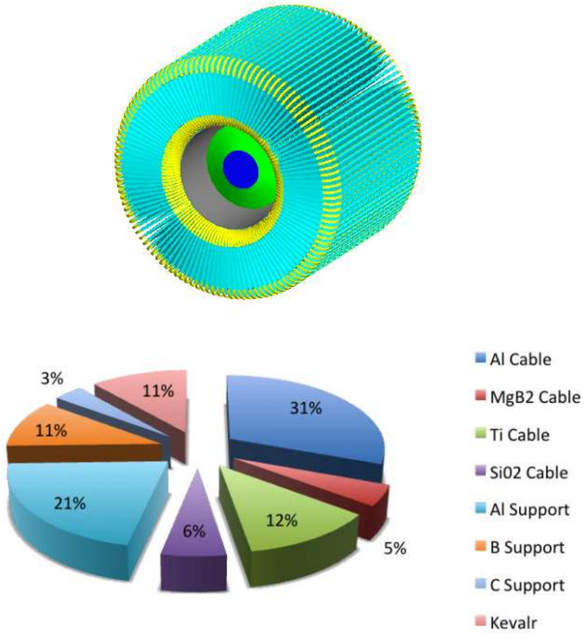

Figure S 3: The material composition of the 8 Tm, continuous-coil toroid shield, including support structures, in the Geant4 simulation. The total mass of the 8 Tm shield is 95.8 t.

2. Tables

Table S 1: List of the structural elements in the 6+1 extendable solenoid shield simulation. The quoted total mass includes the six shield solenoids (33 546 kg), the compensation solenoid and the aluminum shell of the habitat.

element	no.	material	thickness (mm)	mass (kg)
shield solenoid				
coil	1	Cu	0.111	500
support cylinder	1	graphite	10	2 763
radial plate	6	graphite	2.5	388
total mass 5 591 kg				
compensation solenoid				
coil	1	Cu	0.111	400
support cylinder	1	graphite	24	2 132
total mass 2 532 kg				
habitat	1	Al	18	10 136
total mass in the simulation 46 214 kg				

Table S 2: List of the structural elements included in the simulation for the NEA spacecraft chemical propulsion configuration.

element	no.	material	thickness (mm)	volume (m^3)	mass (kg)
re-entry vehicle and access tube					
vehicle structure	1	Al	18		8 868
propulsion	2	liquid methane	-	1.62	2 153
propulsion	2	liquid oxygen	-	0.96	3 435
tube / hatch	1	Al	18	-	4 752
total mass 19 208 kg					
liquid hydrogen container	1	Al	18	-	16 766
liquid oxygen container	1	Al	18	-	4 604
tubular structures	2	Al	18	-	885
total mass 22 255 kg					
total spacecraft mass (excluding habitat and 6+1 shield) 41 463 kg					

Table S 3: Material composition of the NIAC Phase II habitat. The chemical composition of carbohydrates, lipids and proteins is used for the food; the average density used is 0.700 g/cm^3.

element	no.	material	thickness (mm)	volume (m^3)	mass (kg)
cylindrical wall	1	Al	40	-	20 376
end plates	2	Al	18	-	5 582
food	1	$C_5H_{10}O_4$ + N	-	7.8	5 445
water	1	H_2O	-	3.6	3 628
total mass 35 392 kg					

Table S 4: The Geant3 annual GCR dose equivalents (cSv/y) at solar minimum, for the NIAC Phase I and II habitats. The quoted uncertainties represent the root-mean-square deviation of the average dose recorded in the six water cylinders. The Phase II habitat dose estimates were obtained with 300 M protons and He nuclei, and 150 M, Z < 2 nuclei.

**Z**	**Phase I Habitat**			**Phase II Habitat**		
	skin	BFO	body	skin	BFO	body
1	13.1 ± 0.4	12.3 ± 0.2	12.3 ± 0.2	15.2 ± 0.5	14.0 ± 0.4	13.8 ± 0.3
2	6.3 ± 0.3	5.5 ± 0.3	5.4 ± 0.1	6.9 ± 0.7	5.7 ± 0.2	5.8 ± 0.2
3-10	25.7 ± 1.3	16.5 ± 1.1	8.4 ± 0.4	16.8 ± 2.1	11.3 ± 1.0	5.8 ± 0.6
11-20	22.1 ± 1.3	10.6 ± 0.6	8.9 ± 0.5	12.9 ± 1.8	6.8 ± 1.0	5.2 ± 0.7
21-28	11.0 ± 1.6	3.6 ± 0.8	3.8 ± 0.6	5.1 ± 1.1	1.8 ± 0.4	1.7 ± 0.3
**total**	78.2 ± 2.5	48.5 ± 1.5	38.8 ± 0.9	56.9 ± 3.1	39.6 ± 1.5	32.3 ± 1.0
**fraction of Phase I Habitat dose**				0.73 ± 0.05	0.82 ± 0.04	0.83 ± 0.03
